# Revealing fitness and virulence determinants of hypervirulent *Klebsiella pneumoniae* during infection in *Galleria mellonella* using a transposon library

**DOI:** 10.3389/fcimb.2025.1643224

**Published:** 2025-08-22

**Authors:** Lisheng Xiao, Zihan Nie, Deyi Zhuang, Yufeng Zhou, Weiwei Zhu

**Affiliations:** ^1^ Fujian Key Laboratory of Neonatal Diseases, Xiamen Key Laboratory of Neonatal Diseases, Xiamen Children’s Hospital (Children’s Hospital of Fudan University at Xiamen), Xiamen, China; ^2^ State Key Laboratory of Vaccines for Infectious Diseases, Xiang-An Biomedicine Laboratory, National Innovation Platform for Industry-Education Integration in Vaccine Research, Department of Laboratory Medicine, School of Public Health, Xiamen University, Xiamen, China

**Keywords:** *Klebsiella pneumoniae*, virulence, fitness, Tn library, *G. mellonella* infection model

## Abstract

*Klebsiella pneumoniae* infections represent a significant public health concern. Despite their clinical relevance, the genetic determinants underlying bacterial fitness and virulence remain incompletely characterized. In this study, we systematically identified genes involved in host adaptation by generating a transposon mutant library and integrating a *Galleria mellonella* infection model with transposon sequencing (Tn-seq) technology. This approach yielded a comprehensive dataset of adaptation-deficient genes in the hypervirulent *K. pneumoniae* strain ATCC 43816. Using homologous recombination, we constructed gene deletion mutants of the carbohydrate phosphotransferase system enzyme I (PtsI) and the putative prolyl endopeptidase (GM2628), and verified their key roles in *K. pneumoniae* fitness and virulence through both *in vitro* and *in vivo* assays. In particular, *ptsI* defects exhibited lower dissemination and virulence in a murine pneumonia model, which cross-validates that the virulence determinants identified by the *G. mellonella* model are conserved across hosts. Our findings provide gene-level insights for the development of novel strategies to combat *K. pneumoniae* infections and indicate that *G. mellonella* is a cost-effective mammalian alternative for investigating bacterial pathogenicity. Going beyond the general knowledge that hypermucoviscosity (HMV) mediates high virulence, we observed that deficits in *ptsI* and *GM2628* led to HMV while decreasing virulence. This exemplifies that HMV does not always directly correlate with virulence, challenging its role as a virulence marker and underscoring the need for further investigation into non-HMV-mediated virulence mechanisms.

## Introduction

*Klebsiella pneumoniae* (Kp), a Gram-negative opportunistic pathogen, primarily infects newborns, the elderly, and immunocompromised individuals in healthcare settings. With the rise of hypervirulent *K. pneumoniae* (hvKp) and reports of multidrug-resistant hvKp, it has been recognized as a serious threat to public health (Magill et al., 2014; Lan et al., 2021). In addition to directly colonizing hosts, Kp demonstrates a remarkable ability to form persistent biofilms on the surfaces of indwelling medical devices, which makes patients highly susceptible to hard-to-treat healthcare-associated infections (HAIs), such as catheter-associated urinary tract infections and ventilator-associated pneumonia (Paczosa and Mecsas, 2016). However, two major challenges exist in controlling Kp infections. First, the bacterium’s natural resistance greatly undermines the effectiveness of antimicrobial therapies (Holt et al., 2015). Second, the extensive phenotypic plasticity and genetic diversity of Kp allows it to adapt to diverse ecological niches and host environments (Wong Fok Lung et al., 2022). Thus, precisely targeting the adaptive diversity of Kp may be one effective way to control its infection. Unfortunately, a thorough understanding of the determinants of Kp adaptive diversity is still lacking.

The main virulence factors of Kp include capsular polysaccharides (CPS), lipopolysaccharides (LPS), fimbriae, and siderophores (Zhu et al., 2021; Mendes et al., 2023; Tao et al., 2024). CPS are the main virulence factors of Kp and play a critical role in the pathogenic process. However, the sheer diversity of CPS, with over 100 species reported, makes it challenging to find universal treatment or prevention strategies (Shu et al., 2009). The antibody response to microbial glycans, including LPS, often exhibits low affinity and lacks specificity (Rollenske et al., 2018). Therefore, developing new therapeutic targets for Kp necessitates identifying novel pathogenicity factors. Changes in the Kp bacterial load after infection and colonization are linked to adaptability and the capacity to occupy a niche. To gain a clearer understanding of the infection process and the pathogen’s pathogenicity, it is crucial to describe this adaptive shift.

Transposon insertion sequencing (Tn-Seq) is a powerful functional genomics tool that enables the large-scale screening of bacterial gene libraries across the entire genome under simulated environmental stresses, such as immune attacks and nutritional limitations. This process accurately identifies genes that are essential for bacterial survival, colonization, and pathogenicity during infection. This technology has been applied to the investigation of common hospital-acquired bacteria, including *Escherichia coli* (Basta et al., 2025), *Pseudomonas aeruginosa* (Gallagher et al., 2011), *Acinetobacter baumannii*, and *Staphylococcus aureus* (Li et al., 2022). Tn-seq has also been used to identify the determinants of Kp required for lung infection (Bachman et al., 2015), resistance to antimicrobial activities of neutrophils in mice (Paczosa et al., 2020), and growth in human urine and serum (Gray et al., 2024).

*Galleria mellonella* has an innate immune system similar to that of mammals and can survive at temperatures (37°C) where Kp exhibits virulence, making it an excellent model host for infection. This study developed a transposon library in HvKp ATCC 43816 and screened for genes that undergo adaptive changes in the *G. mellonella* infection model through Tn-seq. We assessed the potential of the *G. mellonella* model for investigating bacterial adaptability, identified key adaptive genes, and revealed that these genes also influence bacterial virulence. This adaptive shift is not confined to *G. mellonella*; it is also observed in mammals. Our work accelerates the discovery of virulence factors in HvKp. The generated dataset on fitness decline offers a valuable opportunity to develop antimicrobial therapies targeting weakened pathogens, beginning with early colonization interventions, thus advancing the development of precision antimicrobial therapies.

## Materials and methods

### Strains and growth conditions

The strains, plasmids, and oligonucleotide primers used in this study are listed in **Supplementary Table 1**. *E. coli* and *K. pneumoniae* strains were cultured in LB medium at 37°C on a shaker at 200 rpm. The following antibiotics were added to agar plates or liquid medium as needed: kanamycin (Kan, 50 μg/mL), chloramphenicol (Cm, 34 μg/mL), and ampicillin (Amp, 100 μg/mL).

### Construction of a mariner transposon mutant library in *K. pneumoniae* ATCC 43816

To enable genome-wide functional screening, we constructed a high-density Mariner transposon library in Kp ATCC 43816. This was achieved through biparental conjugation using the suicide vector pKana_Mariner, following established protocols (Paczosa et al., 2020). Briefly, the diaminopimelic acid (DAP)-auxotrophic *E. coli* donor strain WM3064 (OD_600_= 0.5) and the Kp recipient (OD_600_ = 1.0) were harvested, combined at a donor-to-recipient ratio of 15:1 (300 μL:20 μL), and concentrated onto a 0.45-μm filter membrane. The membrane was placed on LB plates supplemented with DAP (57 μg/mL) and incubated at 37°C for 18 h to allow conjugation. Transconjugants were then selected on kanamycin-containing plates, resulting in a library of approximately 60,000 unique mutants. The library was preserved in 50% glycerol at −80°C.

### *G. mellonella* larvae preparation and infection

The *G. mellonella* larvae were obtained from Ruiqingbait (Chongqing, China). Prior to infection, the larvae were surface disinfected with 75% ethanol (v/v). Bacterial suspensions for infection were prepared from overnight cultures of Kp, which were subcultured at a ratio of 1:100 in fresh LB broth. The cultures were grown to OD_600_ = 1.0, harvested by centrifugation, and washed twice with sterile saline. Each larva was then injected with 10 μL of the bacterial suspension, delivering approximately 10^5^ CFU. To distinguish mortality due to infection from trauma induced by the injection procedure, a control group injected with 10 μL of sterile saline was included in all experiments. Following infection, the larvae were incubated in the dark at 37°C and monitored daily for survival over a seven-day period (Insua et al, 2013; Gebhardt et al., 2015; Bruchmann et al., 2021). Larvae were considered dead if they exhibited no response to physical stimuli or characteristic black pigmentation, and the time of death was recorded.

### Tn-seq sample preparation: *in vivo* and *in vitro* DNA isolation

The Kp transposon-mutagenized library was cultured in LB medium containing Kan until OD_600_ = 0.95. Then, a bacterial suspension containing 1 × 10^6^ CFU was injected into individual *G. mellonella* larvae (250–350 mg). Four hours after infection, the larvae were homogenized, and the larval homogenate was uniformly plated onto LB agar supplemented with Kan and Amp. Amp selectively inhibited the native microbiota of *G. mellonella*. The plates were then incubated at 37°C for four hours to allow for bacterial recovery, after which all bacterial cells were collected (*in vivo* output pool).

Concurrently, an *in vitro* control pool was generated by inoculating with the same infection dose in LB medium and incubated at 37°C for four and 24 hours. Bacterial cells from the *in vitro* cultures were harvested by centrifugation. The bacterial cells were then collected via centrifugation (*in vitro* group), and genomic DNA was extracted using a genomic DNA extraction kit (TIANGEN Biotech, China; cat# DP302). Library preparation and analysis were conducted using the previously described barcoded Tn-seq method (Van Opijnen and Camilli, 2012) (**Supplementary Figure 1**). The library preparation and sequencing were performed on the Illumina HiSeq PE150 platform (BGI Genomics, Beijing, China).

### Bioinformatics analysis of Tn-seq data

Tn-seq data analysis was performed as previously described (Van Opijnen and Camilli, 2012). After Illumina sequencing, the raw reads were sorted into groups based on their barcodes using Fastp (Chen et al., 2018). Sixteen-nucleotide fragments from each read, corresponding to the ATCC 43816 genomic sequence flanking the transposon, were mapped to the ATCC 43816 genome (Accession Number: CP194565) using Bowtie 2 (Van Opijnen and Camilli, 2012). The gene IDs mentioned in the text are original sequencing IDs, which may be reprocessed when uploaded to the database. The correspondence between IDs can be found in the **Supplementary Excel File 1**. To identify significant fitness genes in *G. mellonella* and LB medium, fitness values were calculated as log_2_ [(post-infection reads + 1)/(pre-infection reads + 1)], and a Z-test was applied to all gene mutants whose log-transformed output-to-input ratios were significantly different from the overall distribution across all biological replicates. To minimize false-negative results at insertion sites, genes with fewer than two unique insertion sites or fewer than three counts per million reads were excluded from subsequent analyses. Consequently, only genes with a *q*-value less than 0.01 from the Z-test were considered significantly altered from the input (Gao et al., 2017a). For the complete Tn-Seq dataset, see the **Supplementary Excel File 2**.

### Generation and complementation of mutant strains

Gene knockout mutants were generated in ATCC 43816 using allelic replacement via the pDS132 suicide vector system. Flanking regions (~1 kb) of target open reading frames and a kanamycin resistance cassette were PCR-amplified with primers (**Supplementary Table 1**) and cloned into pDS132. Recombinant plasmids were introduced into ATCC 43816 via biparental conjugation with *E. col* S17-1 λpir. Double-crossover recombinants were selected on LB agar supplemented with Kan and 10% (w/v) sucrose, utilizing *sacB*-mediated counter-selection to eliminate the chloramphenicol-resistant plasmid backbone. Transformants grown on Kan-containing LB plates but not on Cm-containing LB plates were isolated, and allelic replacements were confirmed by colony PCR. The genomic fragments containing the coding sequence were cloned into the low-copy vector pACYC184 via the *BamH*I/*Sph*I restriction sites. The recombinant plasmids were then electroporated into the respective mutant strains. Transformants were selected using Cm selection and verified via PCR and sequencing (**Supplementary Table 1**).

### *In vitro* and *in vivo* growth kinetics

*In vitro* growth kinetics: Overnight Kp cultures were diluted 1:100 into 7 mL fresh LB medium and incubated at 37°C. Bacterial growth was monitored hourly by quantifying viable counts (CFU/mL) via plating serial dilutions onto LB agar. *In vivo* bacterial load: To quantify bacterial proliferation within the host, infected larvae were homogenized individually at two time points: at the time of infection (t = 0 hours) and four hours post-infection (t = 4 hours). The homogenates were suspended in 2 mL of sterile saline. The homogenates were serially diluted and plated onto LB agar supplemented with 5 μg/mL chloramphenicol, which selectively recovers Kpn while suppressing the growth of the *G. mellonella* microbiota. Bacterial growth *in vivo* was expressed as the ratio of CFUs per larva at t = 4 hours relative to CFUs per larva at t = 0 hours.

### Competition experiments *in vitro* and *in vivo*

Overnight bacterial cultures were diluted 1:100 into fresh LB broth and incubated to OD_600_ = 1.0. The mutant and WT cells were then mixed at a 1:1 CFU ratio. Serial dilutions were then performed to determine the initial inoculum densities (CFU/mL) for each strain. The mixed culture was diluted 1:100 into 5 mL of fresh LB medium, then co-cultured competitively at 37°C with shaking at 200 rpm for four hours. Post-incubation, the cultures were diluted with saline and then plated separately on antibiotic-free LB plates to count the total CFUs and on Kan-containing LB plates to determine the mutant CFUs. The WT CFUs were calculated by subtracting the mutant CFUs from the total CFUs. Additionally, in the *in vivo* competition assay, 10 µL of the saline-washed mixture (input) was infected with the *G. mellonella*, incubated at 37°C for 4 hours, and then diluted after homogenization before inoculating onto 5 μg/mL Cm-containing LB plates for screen Kp (total output CFUs), and onto Kan- and Cm-containing plates to assess the mutant CFUs (mutant output CFUs). Competition index (CI) = (mutant_output_ CFU/WT_output_ CFU)/(mutant_input_ CFU/WT_input_ CFU) (Gao et al., 2017a).

### Hypermucoviscosity assay

Hypermucoviscosity sedimentation assays were prepared as previously described (Mike et al., 2021). Briefly, the OD_600_ value of the overnight bacterial cultures was measured. Then, 1 mL of the cultures was centrifuged at 1,000 × g for 5 minutes to pellet cells, and 500 μL of supernatant was collected for OD_600_ measurement. The hypermucoviscosity sedimentation assay is evaluated by calculating the ratio of the OD_600_ value of the supernatant to that of the overnight bacterial cultures.

### Murine pneumonia model

Animal experiment protocols were approved by the Institutional Animal Ethics Committee of Xiamen University (Approval Number: XMULAC20190029). Bacteria incubated overnight were diluted 1:100 and reincubated to OD_600_ = 1.0. The bacteria were then centrifuged at 8,000× g for three minutes, and the pellet was resuspended in sterile PBS. 20 g female ICR mice (Charles River Laboratories) were anesthetized via intraperitoneal injection of sodium pentobarbital and subsequently infected intranasally with 1 × 10^5^ CFU of Kp bacteria. 24 hours later, the mice were euthanized by spinal dislocation. Each gram of tissue was homogenized with 9 mL of sterile PBS, spread on plates for counting, and the CFUs were determined. In the competitive experiment, the WT and mutant strains were combined at a 1:1 CFU ratio and infected for 24 hours. The homogenized lung tissue was serially diluted in saline and inoculated onto antibiotic-free LB plates and Kan-containing LB plates. The plates were incubated at 37°C overnight, and the CFUs were counted. The competition index (CI) was calculated using the previously mentioned method, and the detection limit was set at 100 CFU/mL. Samples with no detectable CFUs were assumed to contain 99 CFUs/mL for analysis. Mortality was monitored at 24-hour intervals for 7 days following Kp infection to assess murine survival rates.

## Results

### Construction and evaluation of a high-density transposon mutation library in *K. pneumoniae*

We constructed a Mariner-based transposon insertion library in *K. pneumoniae* ATCC 43816 using a method previously applied to create *Campylobacter jejuni* mutant libraries (Gao et al., 2017a). We assessed the quality of the mutation library through Tn-seq analysis (**Supplementary Figure 1**). A total of 49,786 unique insertion sites were identified across the entire ATCC43816 genome (**Supplementary Table 2**). Next, we conducted a comprehensive analysis of the Tn-seq data to identify genetic determinants associated with infection by *G. mellonella*.

### Screening and analysis of fitness determinants necessary for *K. pneumoniae* infection in *G. mellonella*

**Figure 1:**
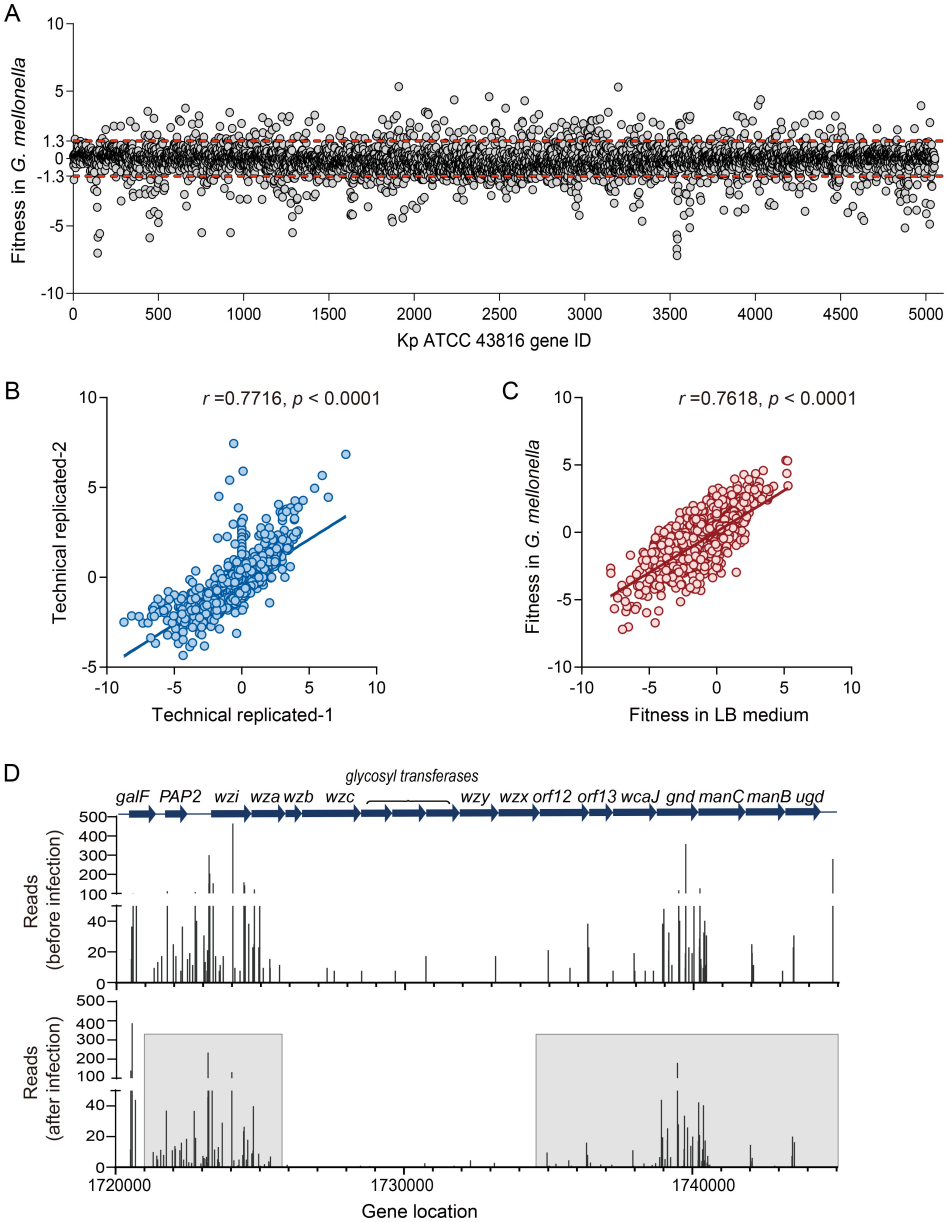
*G. mellonella*-based screening for adaptive gene changes. **(A)** The fitness changes in individual gene insertion mutants of ATCC 43816 after *G. mellonella* infection are shown in a scatter plot. Each point represents the fitness value of a specific transposon insertion mutant. Fitness = 0, indicates no change. The red dashed line marks the thresholds for a ≥4-fold reads change in abundance (fitness value= ± 1.3). **(B)** Reproducibility of experimental protocols. Technical replicates were prepared and sequenced from a single transposon mutant population. Each point represents the fitness numbers of a single gene. The Pearson r value is 0.7716. **(C)** Relative fitness abundances of insertion mutants change *in vivo* and *in vitro*. The relative abundances of mutations in each gene (points) in the LB medium (*in vitro*) and *G. mellonella* (*in vivo*) populations were compared. The Pearson r value is 0.7618. **(D)** The reads of the CPS synthesis genes change in the input (before infection) and output (after infection). The CPS synthesis gene reads decreased after *G. mellonella* infection in the gray boxes.

The *G. mellonella* model is a dose- and time-dependent animal model (**Supplementary Figure 2**). *G. mellonella* were infected with a dose of 10^6^ CFU of Kp per larva at 37°C, as this dose resulted in 50% survival of the larvae after four hours of infection (**Supplementary Figure 2**). Four hours post-infection, the larvae were recovered, homogenized, plated, and cultured for 24 hours to prepare Tn-seq DNA samples. We performed high-throughput sequencing to assess genome-wide adaptive changes in Kp genome four hours post-infection. As shown in **Figure 1A**, each point in the scatter plot represents the calculated fitness value for a specific gene insertion mutant. This fitness value is defined as log_2_ [(post-infection reads + 1)/(pre-infection reads + 1)] and quantifies the relative change in mutant abundance after infection compared to the pre-infection baseline. The key observation is the significant dispersion of points away from the central baseline (fitness = 0, indicating no change, **Figure 1A**). We defined reads change exceeding a 4-fold increase or decrease (corresponding to fitness value = ± 1.3) as indicative of a biologically significant adaptive change. The high density of points falling outside the |fitness| = 1.3 threshold (both above and below zero) across numerous gene IDs provides direct visual evidence of widespread adaptive changes in the Kp genome four hours post-infection. Furthermore, the reproducibility of the Tn-seq results across biological replicates was confirmed (Pearson correlation *r* = 0.7716, **Figure 1B**).

*In vitro*, equal amounts of mutant libraries were inoculated in LB medium and collected after 4 and 24 hours of incubation. We found that bacteria undergoing adaptive changes in *G. mellonella* exhibit similar correlations in LB medium (*r* = 0.7618, **Figure 1C**), indicating that genes essential for proliferation *in vitro* often overlap with adaptive requirements *in vivo* (Ibberson et al., 2017). The reliability of the model in identifying adaptive and virulence factors is supported by the reduction in the reads of genes in the CPS biosynthesis operon post-infection, accompanied by a decrease in fitness values (Z-test, *p* < 0.01) (**Figure 1D**, **Supplementary Table 3**). The connection between bacterial adaptation and the retention of virulence factors further demonstrates the strength of this model in clarifying the trade-off between pathogenicity and adaptability. Tn-seq analysis revealed genes with low insertion density, such as *wzb*, *wza*, and *wzy*, which were attributed to transposase insertion bias (**Figure 1D**). To avoid false negative results at insertion sites, genes with fewer than two unique insertion sites were excluded from subsequent analyses.

**Figure 2:**
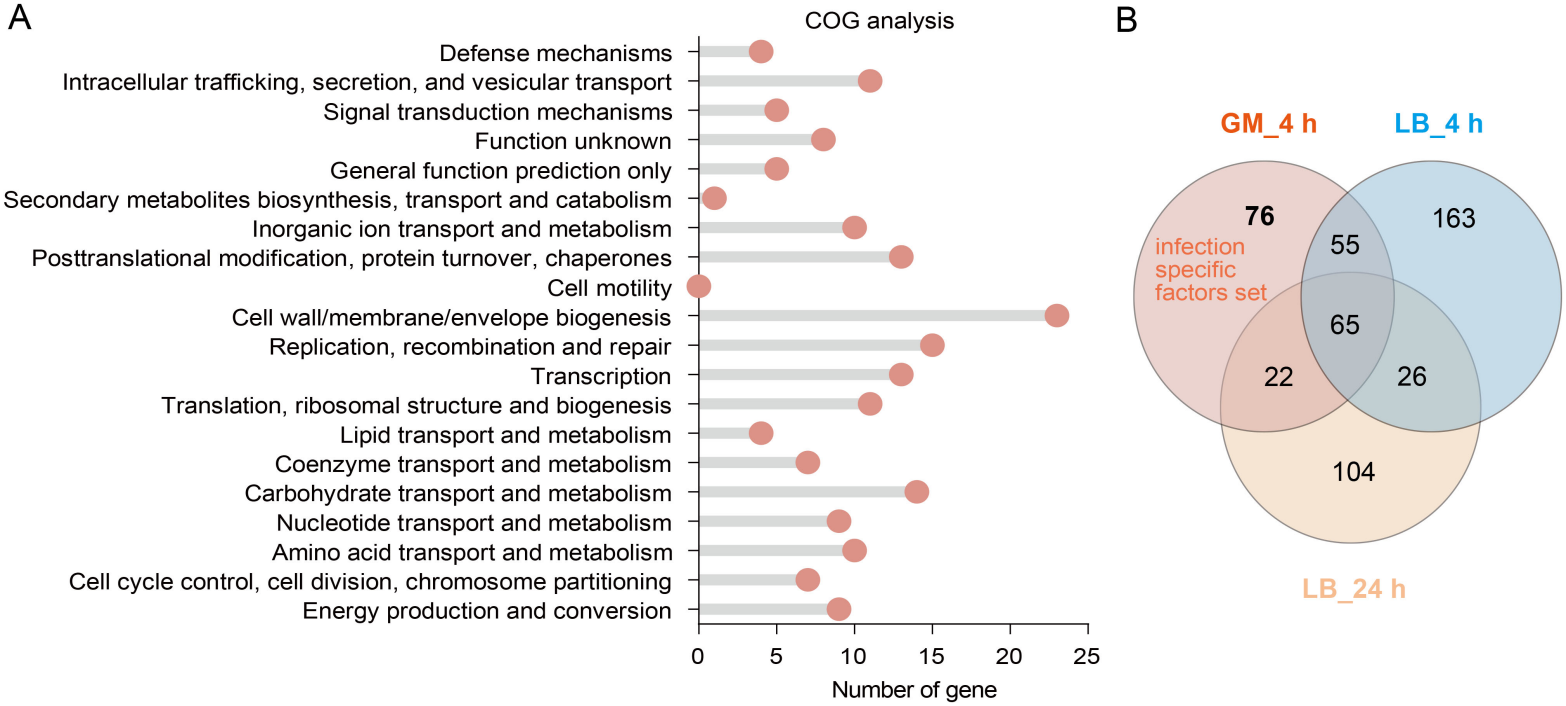
Functional analysis of Kp fitness-defective genes in *G. mellonella*. **(A)** COG functional enrichment analysis of fitness-defective genes after *G. mellonella* infection (*p*< 0.01). **(B)** A Venn diagram identifying infection-specific essential genes. GM_4 h: Fitness-defective genes after a 4 hours infection in *G. mellonella*. LB_4 h and LB_24 h: Fitness-defective genes after 4 hours and 24 hours of incubation in LB medium, respectively. The Arabic numerals in the circles indicate the number of genes.

To systematically characterize the biological functions underlying bacterial adaptation *in vivo*, we performed a Clusters of Orthologous Groups (COG) enrichment analysis on adaptive-related genes identified in *G. mellonella* (Padj < 0.01). This approach enabled us to map these genes to conserve functional categories and pinpoint critical pathways for host adaptation. COG enrichment analysis revealed that adaptive-defect genes significantly enriched in specific COG functional terms compared with the input database (**Figure 2A**, **Supplementary Table 3**). Notably, these genes were enriched in the functional categories of “cell wall/membrane/envelope biosynthesis,” “replication, recombination, and repair,” and “carbon source transport and metabolism.” These results demonstrate that structural integrity, via cell envelope biogenesis, and metabolic flexibility are key adaptive strategies employed by Kp during *G. mellonella* infection.

To distinguish genes specifically required for infection from those essential for general bacterial growth, we conducted a Venn analysis comparing fitness-defective genes (*p* < 0.01) *in vivo* (*G. mellonella*) versus *in vitro* (LB medium). This comparative design aimed to identify infection-specific vulnerabilities that could serve as targets for therapeutic intervention. The Venn analysis identified 76 infection-specific genes (**Figure 2B**). Of these, 86.2% (50 out of 58) have well-established roles in the pathogenicity of bacteria (including Kp) (excluding 18 uncharacterized ORFs; **Supplementary Table 3**, gray background). This high proportion of known virulence-associated genes within this subset validates our model’s ability to uncover genuine host adaptation mechanisms and highlights potential targets for disrupting infection-specific pathways.

### Validation of candidate fitness factors in *G. mellonella* infection

**Figure 3:**
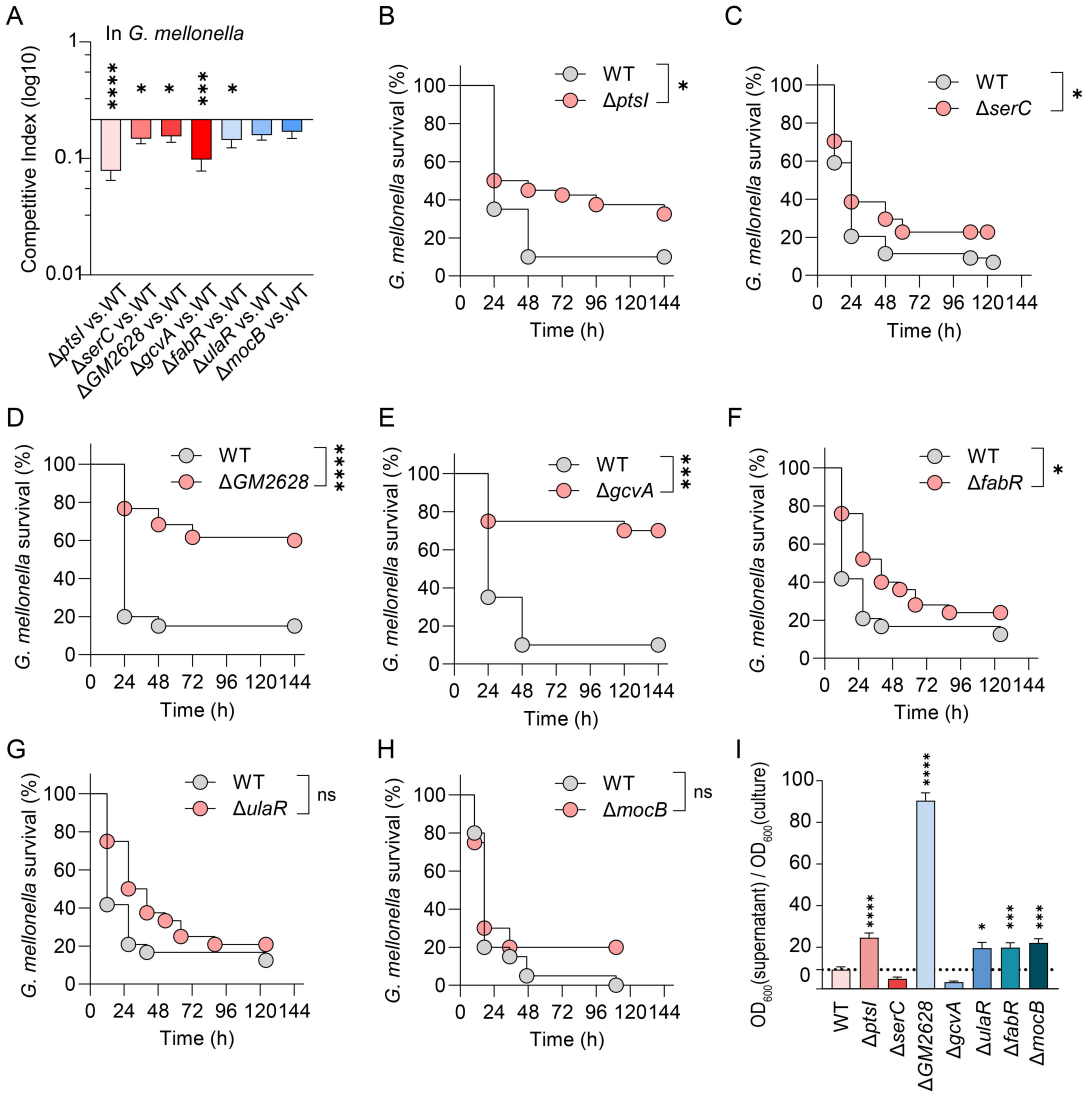
Fitness-deficient genes exhibit significantly attenuated virulence in *G. mellonella.* **(A)** Competitive survival assay in *G. mellonella* model. The competition index was measured after the WT and mutant strains were infected with a CFU ratio of 1:1 for 4 h (n=7). **(B-H)** Survival curve in *G. mellonella*. 10^5^ CFU WT and mutant strains infected *G. mellonella* (n=12-24). **(I)** Hypermucoviscosity assay. Following centrifugation at 1000 g for 5 min, the ratio of OD_600_ in supernatant to OD_600_ before centrifugation was calculated to determine viscosity. The error bars represent the mean ± standard error of the mean (SEM). Unpaired *t*-test, *p < 0.05; ***p < 0.001; ****p < 0.0001.

To identify novel virulence factors for bacterial survival during infection, we validated seven candidate genes associated with adaptive costs (**Table 1**), based on transposon libraries obtained. Growth curves indicated that deleting these seven genes alone did not affect the growth rate of Kp in LB medium (**Supplementary Figure 3**). However, when WT and mutant strains were inoculated at a 1:1 CFU ratio into LB medium for a competitive growth experiment *in vitro*, the Δ*ptsI* and Δ*GM2628* mutant strains displayed severe adaptive (mean CI = 0.09) and moderate adaptive defects (mean CI = 0.275) (**Supplementary Figure 4A**), which were reversed by *ptsI* and *GM2628* genetic complementation (**Supplementary Figure 4B**). These results indicate that PtsI and GM2628 are critical for the rapid proliferation of Kp under *G. mellonella* host conditions. Competitive fitness assays *in vivo* revealed that five mutant strains (Δ*ptsI*, Δ*serC*, Δ*GM2628*, Δ*gcvA, and* Δ*fabR*) exhibited significant adaptive defects compared to the WT in *G. mellonella (* **Figure 3A**). In contrast, deletion of *ulaR* or *mocB* had no effect on competitive fitness *in vivo* (**Figure 3A**). Consistently, *G. mellonella* infection experiments demonstrated that the same five mutants displayed attenuated virulence, as evidenced by increased larval survival (**Figures 3B-F**, **Supplementary Figure 5**). Similar to the competitive fitness results, *ulaR* and *mocB* deletions did not significantly impact virulence (**Figures 3A, G, H**). By linking these genes to their functions, this study uncovered that carbohydrate metabolism (*ptsI*), amino acid metabolism (*serC*, and *gcvA*), and fatty acid biosynthesis (*fabR*) are determinants of survival and adaptability in host-pathogen interactions. These findings underscore the importance of metabolic flexibility as a vital survival strategy during host adaptation.

**Table 1:** Characteristics of the candidate genes and their fitness value in different treatments^a^.

Gene id	Gene	Product/Function	Average fitness value (GM_4 h)	Average fitness value (LB_4 h)	Average fitness value (LB_24 h)
GM001369	*ptsI*	Carbohydrate transport	-2.74	-4.99	-6.08
GM003320	*serC*	Serine biosynthesis	-3.35	-0.29	-0.33
GM002628	*GM2628*	Putative proly endopeptidase	-1.88	-5.22	0.47
GM000977	*gcvA*	Repressor of the glycine cleavage enzyme system	-3.00	-2.41	-0.51
GM004609	*ulaR*	Repressor of L-ascorbic acid transport and catabolism.	-2.41	-2.19	-0.97
GM004925	*fabR*	Repressor fatty acid biosynthesis	-2.95	-2.78	-0.05
GM002001	*mocB*	Capsular Polysaccharide biosynthesis	-1.35	-0.05	-0.05

a: Fitness values represent log_2_ [(post-infection reads + 1)/(pre-infection reads + 1)]. GM_4 h indicates 4 hours of infection in *G. mellonella*. LB_4 h and LB_24 h represent 4 hours and 24 hours of incubation in LB medium, respectively.

### The pathogenicity of hvKp in *G. mellonella* is not dependent on the hypermucoviscosity

To investigate the association between hypermucoviscosity (HMV) and the virulence of hvKp, we employed centrifugation-based assays to quantify HMV. In these assays, viscous cells remain suspended, while non-viscous strains form compact pellets. Our findings revealed that under centrifugation conditions of 1,000 × g, the HMV of the Δ*ptsI*, Δ*GM2628*, Δ*fabR*, Δ*ulaR*, and Δ*mocB* mutant strains was significantly higher than that of the WT strain (**Figure 3I**). However, in the *G. mellonella* infection model, the virulence of the Δ*ptsI*, Δ*GM2628*, and Δ*fabR* mutant strains was significantly diminished, while the virulence of the Δ*ulaR* and Δ*mocB* strains remained unchanged (**Figure 3**). These results suggest that HMV does not directly correlate with enhanced virulence in the *G. mellonella* model.

Further analysis of the relationship between HMV and adaptive defects showed that, although the Δ*serC* and Δ*gcvA* mutants-maintained HMV levels like the WT strain (**Figure 3I**), their adaptability was significantly reduced in *G. mellonella* (**Figure 3A**). In contrast, the Δ*ulaR*, Δ*fabR*, and Δ*mocB* mutants exhibited increased HMV but no enhanced fitness (**Figures 3A, I**). These results suggest that adaptive defects can directly lead to reduced virulence independently of HMV changes. In summary, HMV is not a reliable marker of HvKp virulence in the *G. mellonella* model, and bacterial adaptation may regulate virulence in this model through HMV-independent pathways.

### Validation of fitness costs and virulence in Kp across *G. mellonella* and mouse models

**Figure 4:**
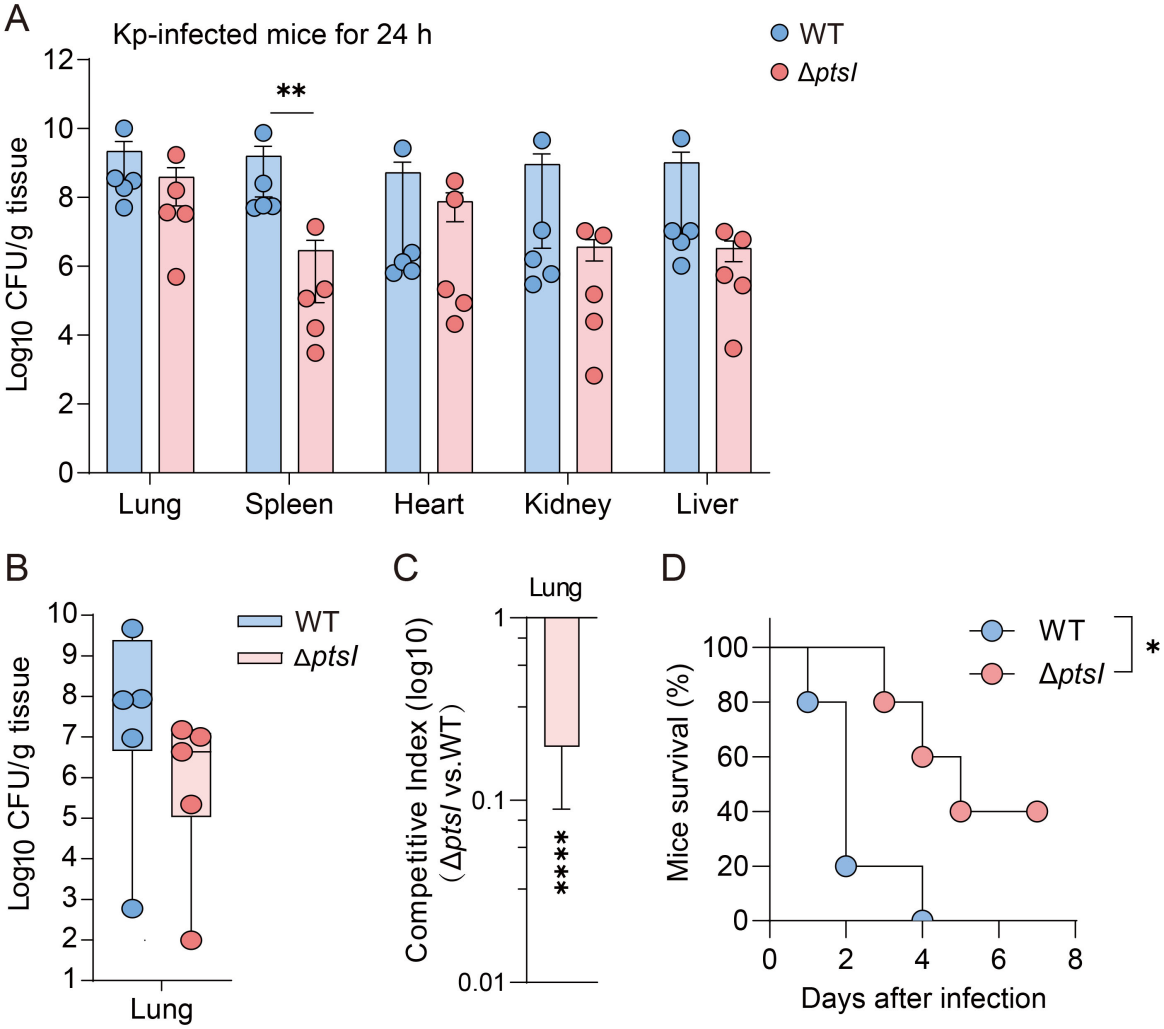
Validate the relationship between fitness and virulence in *G. mellonella*, as well as in murine infection models. **(A)** Bacterial loads in different organs of mice 24 hours post-infection (n=5 per group, Unpaired *t*-test). **(B, C)** Following the 1:1 CFU ratio of mutant and WT infection, CFU/g **(B)** and the competition index **(C)** in lung tissues were measured 24 hours post-infection (n=5 per group, Unpaired *t*-test). **(D)** Survival curves of mice infected with WT or Δ*ptsI* (1.5×10^4^ CFU per mice, n = 6 per group, Log-rank test). The error bars represent the mean ± standard error of the mean (SEM). *p < 0.05; **p < 0.01; ****p < 0.0001.

The *G. mellonella* infection model has become a popular tool for assessing pathogenicity. However, intrinsic physiological differences exist among various host models. Therefore, we investigated whether the fitness cost incurred by Kp genes during *G. mellonella* infection also occur in a mammalian host (mice), and whether fitness cost impact virulence. We used the *K. pneumoniae* mutant strain Δ*ptsI* for cross-model validation. In murine lung infection experiments, mice were inoculated with 1.5 × 10^4^ CFU of WT or Δ*ptsI* strain. The Δ*ptsI* mutant maintained lung bacterial loads comparable to the WT strain 24 hours post-infection, indicating intact pulmonary colonization capacity (**Figure 4A**). However, its splenic bacterial load was significantly reduced (*p* < 0.01), suggesting impaired systemic dissemination (**Figure 4A**). Competitive experiments conducted 24 hours post-infection with a 1:1 CFU ratio of WT and mutant strains revealed differences in bacterial load (**Figure 4B**), with significant fitness defects observed in the lungs (**Figure 4C**). The competitive index in the lungs was 0.2 for Δ*ptsI* (**Figure 4C**). Consistently, Δ*ptsI*-infected mice 40% survival at seven days versus 100% lethality in WT controls (**Figure 4D**), indicating attenuation of virulence in Δ*ptsI*. These results recapitulate the adaptation and virulence defects observed in *G. mellonella*, demonstrating conserved infection dynamics between the insect and murine pneumonia models.

## Discussion

Despite the growing clinical burden posed by hvKp, the molecular mechanisms underlying its pathogenicity are not fully understood. In this study, we systematically identified the adaptive determinants required for infection by combining genome-wide Tn-seq with the *G. mellonella* infection model. This approach revealed 76 infection genes (*p* < 0.01) (**Figure 2B**), whose functions converge on metabolic plasticity as the dominant survival strategy. These genes collectively orchestrate an integrated network that channels metabolic adaptability, envelope biogenesis, nutrient acquisition, stress tolerance and efflux capacity act in concert toward efficient host colonization.

Our findings provide a rational framework for discovering next-generation anti-virulence targets (**Supplementary Table 3**). Disruption of various nodes in this adaptive network in Kp and other pathogenic bacteria recapitulates the attenuated virulence phenotype. The genes *glpD*, *wzi*, *waaQ*, *wabN*, and *bamB* are involved in CPS/LPS synthesis, and mutant strains exhibit reduced pathogenicity (Klein and Raina, 2019). A *galU* or *fepB* mutant displayed reduced adaptability in a mouse model, highlighting the significance of nutrient acquisition and iron metabolism in host colonization (Lai et al., 2001; Palacios et al., 2017). In the *G. mellonella* model, strains lacking pyrimidine (*pyrC*, *pyrD*, *pyrE*, *pyrF*, *nrdB*, *carA*, and *carB*) and purine biosynthesis exhibited significant adaptive defects. This underscores the importance of nucleotide synthesis in nutrient-limited host environments (Jenkins et al., 2011; Sause et al., 2019). Disruptions in the aromatic amino acid (*aroA*, *aroB*, *aroC*, and *aroG*) and vitamin B6 (*pdxJ*, *pdxA*, *pdxH*, and *pdxC*) synthesis pathways reduce Kp survival (Holmes et al., 2023), consistent with previous studies of *Salmonella* and *Mycobacterium* (Dick et al., 2010; Grubman et al., 2010; El Qaidi et al., 2013). Energy-dependent proteases, including the Lon and Clp families, are crucial for Kp virulence. Transposon insertion into the *lon* and *hslV* genes hinders bacterial adaptability, aligning with their roles in degrading misfolded proteins and regulating virulence factors in *Yersinia pestis* and *Salmonella* (Jackson et al., 2004; Farrand et al., 2013; Muselius et al., 2020). These findings suggest that a conserved mechanism exists to coordinate stress responses and pathogenic gene expression through proteases. Role of the AcrAB-TolC efflux system (*acrA*, *acrB*, and *tolC*) in Kp adaptation to *G. mellonella*, possibly by conferring antibiotic resistance and efflux of host-derived antimicrobial peptides (Virlogeux-Payant et al., 2008; Zhang et al., 2021). Additionally, the contribution of ion transport systems (*trkA*, *trkB*, and *znuB*) to Kp survival underscores the importance of potassium and zinc access during infection, which aligns with observations in *A. baumannii* (Gebhardt et al., 2015). Overall, these findings suggest that Kp establishes infection through a multifaceted network reliant on virulence factors, metabolic adaptability, stress response systems, and nutrient acquisition mechanisms. The conservation of these pathways across various pathogens highlights their importance in the evolution of bacterial pathogenicity.

Metabolic defects in the Δ*ptsI*, Δ*serC*, Δ*gcvA*, and Δ*fabR* mutants confirm metabolic remodeling as a necessary compensatory mechanism (**Figure 3**). The metabolic genes we screened are highly conserved in *P. aeruginosa*, S*. aureus*, and a broader range of *Enterobacteriaceae* bacteria (Riquelme et al., 2020; Zhi et al., 2020; Lohia and Riquelme, 2025). This suggests that metabolic reprogramming may serve as a universal anti-pathogenic axis. The metabolic plasticity that enhances pulmonary adaptation in these bacteria highlights the therapeutic potential of reprogramming pathogen metabolism (Wong Fok Lung et al., 2022). However, the mechanisms by which bacteria perceive host signals and translate them into downstream metabolic and regulatory networks remain unclear. Therefore, we will cross-analyze the gene set identified in *G. mellonella* with the gene set exhibiting adaptive changes in mouse lung infections (Bachman et al., 2015) to identify indispensable nodes under different immune environments. Nevertheless, the translational application of these targets must consider trade-offs in fitness costs. While Δ*ptsI* mutant reduce virulence, they also enhance antibiotic tolerance and promote persistent infection in Kp (Parsons et al., 2024). Thus, before advancing anti-bacterial strategies targeting multidrug-resistant pathogens, the entire gene set must be rigorously validated under antibiotic stress conditions.

The HvKP strain is best characterized by its HMV phenotype (Walker and Miller, 2020; Zhu et al., 2022; Wu et al., 2024). However, our findings challenge this notion. Despite its excessive capsule production, the Δ*GM2628* mutant exhibited a significant survival defect in *G. mellonella* model; the same trend was observed in the Δ*ptsI* strain (**Figure 3**). These data suggest that excessive capsular production does not provide protection against host defenses in an innate immune environment dominated by antimicrobial peptides. Therefore, the role of HMV in responding to innate immune processes requires reevaluation. We speculate that HMV may not contribute to bacterial resistance against antimicrobial peptide production. Its specific biological functions require further investigation.

Additionally, our dataset is limited to a single early time point (4 hours) and an innate immunity-only host. Future work should extend these observations to murine models that incorporate adaptive immunity. Additionally, single-cell transcriptomics should be integrated with isotope-traced metabolomics to illuminate how HvKp dynamically balances virulence, adaptability, and antibiotic resistance throughout infection. In the meantime, the Tn-seq–*G. mellonella* platform provides a quick, inexpensive pipeline for the large-scale discovery of pathogenicity determinants across the Enterobacteriaceae.

## Data Availability

The datasets presented in this study can be found in online repositories. The names of the repository/repositories and accession numbers can be found in the article/**Supplementary Material**.
