# Supplementary material for: Revealing fitness and virulence determinants of hypervirulent Klebsiella pneumoniae during infection in Galleria mellonella using a transposon library
Source: Front Cell Infect Microbiol. 2025 Aug 22;15:1643224. doi: 10.3389/fcimb.2025.1643224 (PMC12411516; doi:10.3389/fcimb.2025.1643224)
Supplement: Supplementary file 1 [file DataSheet1.pdf]

Supplementary Material

Supplementary Figures

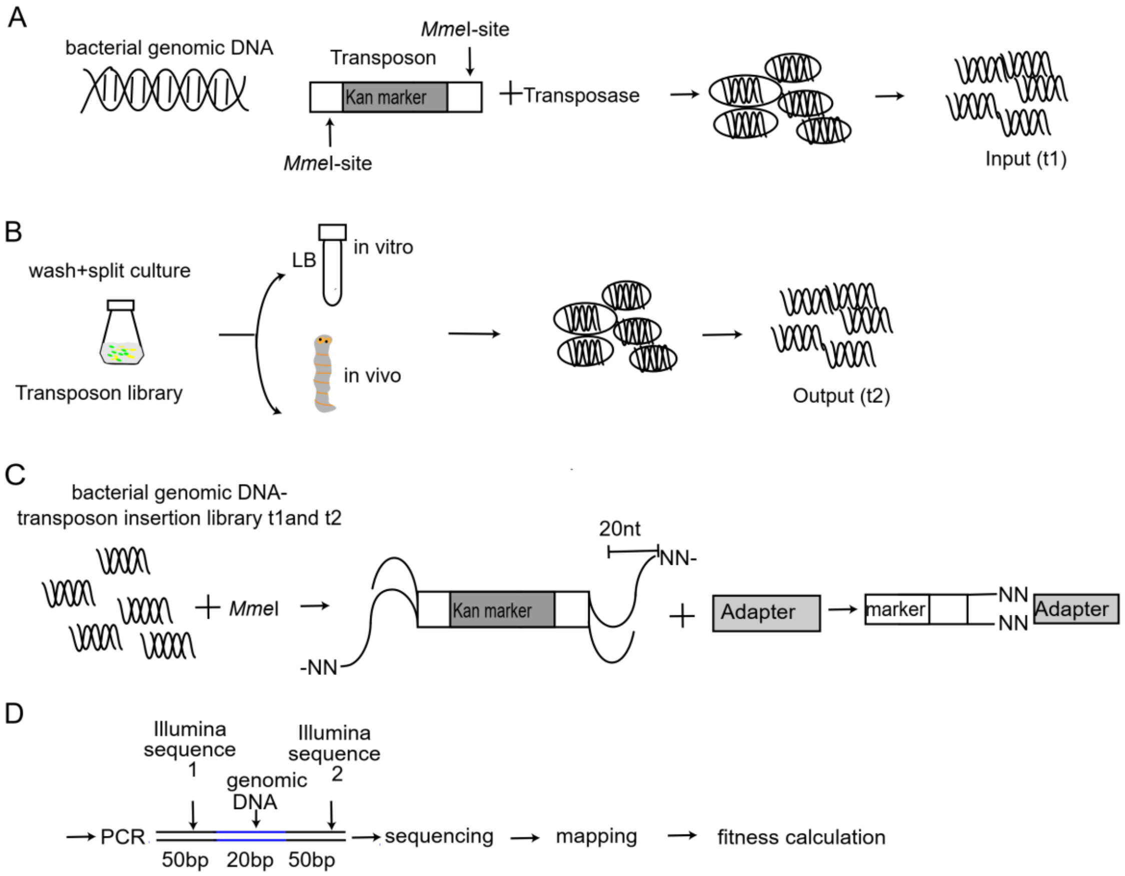

Supplementary Figure 1. Tn-seq scheme. (**A and B**) Gene mutation libraries were constructed by transposing Magellan6 into bacterial genomic DNA in vitro and transforming bacterial populations with the transposed DNA, creating a library of strains in which each bacterium contains a single transposon that has been randomly inserted into its genome. A portion of the bacterial bank was isolated for DNA extraction (input t1), while another portion was used to inoculate selected cultures (*in vitro* or *in vivo*). After selection, the bacteria were recovered, and the DNA was isolated again (output t2). (**C**) The DNA from t1 and t2 was digested with *Mme*I, which binds to the reverse repeat sequence at the end of Magellan6 but cleaves 20 bases downstream, leaving a two-base protrusion where a connector binds. In PCR, one primer was complementary to the connector sequence, and the other was complementary to the reverse repeat sequence. (**D**) A PCR product consisting of 120 bp of bacteria-specific DNA flanked by Illumina-specific sequences for sequencing was generated. Based on the barcode sequence, fitness was calculated by mapping the bacterial readings to the genome and counting the number of insertions.

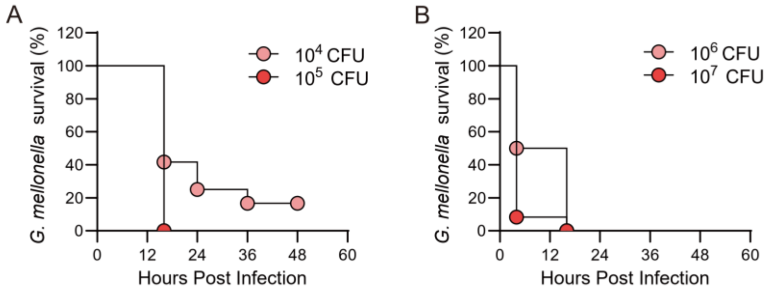

Supplementary Figure 2. Survival of *G. mellonella* post infection with the indicated dose of ATCC 43816 (n=12 per group).

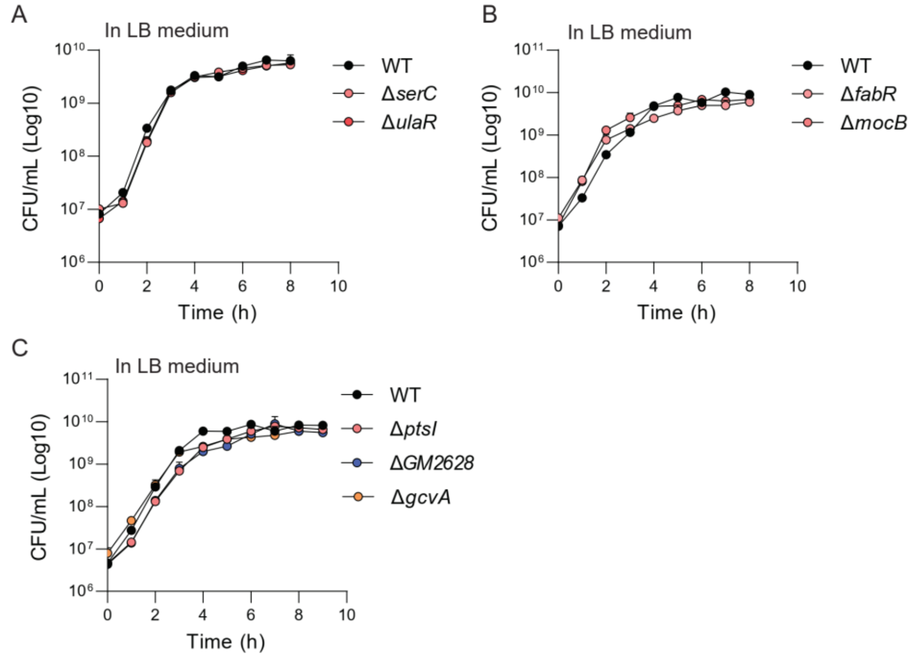

Supplementary Figure 3. Growth curve of Kp strains in LB medium (**A–C**). The error bars represent the mean ± standard error of the mean.

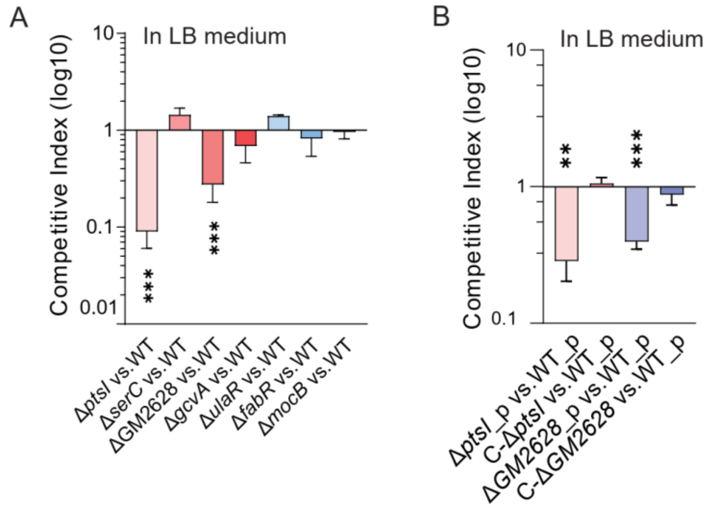

Supplementary Figure 4. Competitive survival assay in LB medium. **(A)** Competitive fitness in LB medium. WT and mutant strains were mixed (1:1 CUF ratio), cultured at 37°C for 4 hours, then plated on Kan-containing or Kan-free LB plates to determine competition indices. (**B**) Competitive fitness in LB medium (complementation assay). CIs were determined by co-culturing WT strains harboring an empty plasmid and mutant strains harboring the complementation plasmid at a 1:1 CFU ratio. The culture treatment method was similar to that shown in panel A. Statistical significance was assessed using unpaired *t*-test, comparing each group to CI = 1 (**$p < 0.01$, ***$p < 0.0001$).

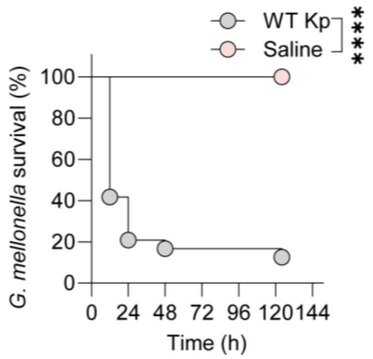

Supplementary Figure 5. Survival of *G. mellonella* post-infection. *G. mellonella* larvae (n=12 per group) were injected with 10^5 CFU of WT strain or 10 µL saline (mock infection control). Significance of survival differences was assessed with the log-rank test (****, $p < 0.0001$).

Supplementary Tables

Supplementary Table 1. Strains, plasmids, and primers used in this study.

Strains	Genotype/Description	Source/Usage
Klebsiella pneumoniae		
ATCC 43816	Wild type	Laboratory stock
Δ*GM2628*	ATCC43816 harboring in-frame deletion of K43816_GM002628, kanamycin resistance	This work
Δ*serC*	ATCC43816 harboring in-frame deletion of K43816_GM003320, kanamycin resistance	This work
Δ*gcvA*	ATCC43816 harboring in-frame deletion of K43816_GM000977, kanamycin resistance	This work
Δ*ulaR*	ATCC43816 harboring in-frame deletion of K43816_GM004609, kanamycin resistance	This work
Δ*fabR*	ATCC43816 harboring in-frame deletion of K43816_GM004925, kanamycin resistance	This work
Δ*ptsI*	ATCC43816 harboring in-frame deletion of K43816_GM001369, kanamycin resistance	This work
Δ*mocB*	ATCC43816 harboring in-frame deletion of K43816_GM002001, kanamycin resistance	This work
C-Δ*ptsI*	ΔGM001369 mutant harboring complementation plasmid pACYC184-*ptsI*, kanamycin and chloramphenicol resistance	This work
C-Δ*GM2628*	ΔGM002628 mutant harboring complementation plasmid pACYC184-*GM2628*, kanamycin and chloramphenicol resistance	This work
WT_p	Wild type harboring empty vector pACYC184, kanamycin and chloramphenicol resistance	This work
Δ*ptsI*_p	ΔGM001369 mutant harboring empty vector pACYC184, kanamycin and chloramphenicol resistance	This work
Δ*GM2628*_p	ΔGM002628 mutant harboring empty vector pACYC184, kanamycin and chloramphenicol resistance	This work
Escherichia coli		
S17-1 λpir		Laboratory stock
WM3064	Auxotrophic to DAP. This strain can be used for conjugation experiments and replication of plasmids that require the pir protein.	Laboratory stock
DH5α	*supE44 ΔlacU169 (φ80lacZΔM15) hsdR17 recA1 endA1 gyrA96 thi-1 relA1*	Laboratory stock
**Plamids**		
pKana_Mariner	This suicide plasmid contains mariner transposase, beta-lactamase, and Kan genes, kanamycin resistance	Laboratory stock
pDS132	This suicide plasmid, chloramphenicol resistance.	Laboratory stock
pKD4	This is a template plasmid for an FRT-flanked Kan cassette, kanamycin resistance.	Laboratory stock
pACYC184	Complementation plasmid, chloramphenicol resistance.	Laboratory stock
pACYC184-ptsI	130 bp upstream + full *ptsI* CDS + 50 bp downstream, cloned into pACYC184 via *BamH*I/*Sph*I. kanamycin and chloramphenicol resistance.	Laboratory stock
pACYC184-2628	336 bp upstream + full *GM2628* CDS + 70 bp downstream, cloned into pACYC184 via *BamH*I/*Sph*I. kanamycin and chloramphenicol resistance.	Laboratory stock

Continued from Supplementary Table 1.

Primers	Sequence（5′--3′）	Usage
kp-2628-1FR	GTGGAATTCCCGGGAGAGCTCGTGCAGGGTTAACAGACCT/ GCAGCTCCAGCCTACACAGCGCATATGACAGAACGACAT	Amplification of homologous fragments
Kana-2FR	TGTGTAGGCTGGAGCTGC/ CATATGAATATCCTCCTTAG	Amplification of homologous fragments
kp-2628-3FR	CTAAGGAGGATATTCATATGATGGCGGCGAGCTGACG/ ACCGCATGCGATATCGAGCTCGCAGACGATCCCAAGGCT	Amplification of homologous fragments
Kp-*ptsI*-1FR	GTGGAATTCCCGGGAGAGCTCAAGTTTTACCCCTCCTGG/ CATAACCCTACCTTACTTGTGA	Amplification of homologous fragments
Kp-*ptsI*-3FR	CTGCTAATCCACGAGATGC/ ACCGCATGCGATATCGAGCTCTCCCGCGAGAAGTGCTAT	Amplification of homologous fragments
kp-*gcvA*-1FR	GTGGAATTCCCGGGAGAGCTCTGGTAGAACCTCAACGGACAA / GCAGCTCCAGCCTACACATGCGGCCTGCGTCACAAA	Amplification of homologous fragments
kp-*gcvA*-3FR	CTAAGGAGGATATTCATATGCGCTTTCGTTATGAACA/ ACCGCATGCGATATCGAGCTCGGCAAAACCAAACACCTC	Amplification of homologous fragments
kp-*serC*-1FR	GTGGAATTCCCGGGAGAGCTCACGCTATACCACCATTCC/ AGCCATTTCCCCTCACCAC	Amplification of homologous fragments
kp-*serC*-2FR	TGGTGAGGGGAAATGGCTTGTGTAGGCTGGAGCTGC/ AGAAACAGGCGATTAACCCATATGAATATCCTCCTTAG	Amplification of Kana fragments
kp-*serC*-3FR	GGTTAATCGCCTGTTTCT/ ACCGCATGCGATATCGAGCTCGCAGGGCATCCACCAGAT	Amplification of homologous fragments
kp-*fabR*-1FR	GTGGAATTCCCGGGAGAGCTCGCTCCCTGTTTCACCAGTC/ GCAGCTCCAGCCTACACATTGTGCTCTTACGCCCATC	Amplification of homologous fragments
kp-*fabR*-3FR	CTAAGGAGGATATTCATATGTAACGTGAAGGACGAGCAATG/ ACCGCATGCGATATCGAGCTCGTAGCCGATCCAGAAGACC	Amplification of homologous fragments
kp-*ulaR*-1FR	GTGGAATTCCCGGGAGAGCTCGGCGATTCCCACTACTCCA/ GCAGCTCCAGCCTACACACCTTTGCGCTTCCGTCAT	Amplification of homologous fragments
kp-*ulaR*-3FR	CTAAGGAGGATATTCATATGGTTTAAAGGTGCTGCTGAAA/ ACCGCATGCGATATCGAGCTCCTCGCCTGATTTTGATGT	Amplification of homologous fragments
kp-*mocB*-1FR	GTGGAATTCCCGGGAGAGCTCCCATCACGCAGTCGTTTAA/ GCAGCTCCAGCCTACACAGTGCTTCATAGTATGCCCTCAG	Amplification of homologous fragments
kp-*mocB*-3FR	CTAAGGAGGATATTCATATGCATTAACGGCAAGGCGCAGGC/ ACCGCATGCGATATCGAGCTCCGCTGGCGGCATTGGTCA	Amplification of homologous fragments
DQ-2628-1FR	GCAGCAACGCACCGACCAGA/ ACACGGCGGCATCAGAGCAG	Check primer
DQ-2628-2FR	GAAATGACCGACCAAGCG/AGCACTACCACCGTAGCAAA	Check primer
CX-43816 *ptsI*-FR	GCCGCCTGATGGAAGAAG/GATAGCCCAGTAGCTGACATT	Check primer
DQ-*gcvA*-1FR	CTTTGCTACCAGGGTCTGC/ CCGATTGTCTGTTGTGCC	Check primer

Continued from Supplementary Table 1.		
DQ-*gcvA*-2FR	ATTCCACCGCCGCCTTCT/ GCAGTTCGCCGACCACAA	Check primer
CX-43816 *serC*-FR	CAGTTGGCCTGGGAGAAG/ CATCAGAGCAGCCGATTGT	Check primer
DQ-*fabR*-1FR	TGGTTGATGACGCTGTCG/ CGTGTTCCGCTTCCTTTA	Check primer
DQ-*fabR*-2FR	ATCAGGACATAGCGTTGGC/ CTCGGCTGCGGTATCTTT	Check primer
DQ-*ulaR*-1FR	GACCCACGATCACAACGA/ CTGCGGACTGGCTTTCTA	Check primer
DQ-*ulaR*-2FR	ATCAGGACATAGCGTTGGC/ GCAGCAGAATTTCAGGGAGT	Check primer
DQ-*mocB*-1FR	CTGACGGTGAGCGAACTGA/ TGCGTGCAATCCATCTTG	Check primer
DQ-*mocB*-2FR	GCGGACCGCTATCAGGACA/ ACCCGGACGACACGCTACTC	Check primer
C-*ptsI*-F/R	CGGGATCCGCACCGTAGTGACCCTCT/ CATGCATGCAAACCCATGATCTTCTCCT	Complementation plasmid construction
C-2628-F/R	CGGGATCCAAGCATAAGCGGCAGCAC/ CATGCATGCCGACGGGAACGATATTTGTA	Complementation plasmid construction
JD-pACYC -F/R	ACTGTCCGACCGCTTTGG/ GGCACCTGTCCTACGAGTTG	Verification of the complementation plasmid

Supplementary Table 2. Insertion site statistics.[a]

Barcode	sample name	Total insertions	Insertions inside of ORF	Insertions located intergenic region
**LB Input**				
TTGG	T0h LB 1	49786	40541	9245
ATCG	T0h LB 2	41283	33716	7567
CACT	T0h LB 3	41442	33703	7739
**LB Output**				
AACT	T4h LB 1	45799	37107	8692
TATA	T4h LB 2	27655	22501	5154
TCAG	T4h LB 3	34953	28392	6561
***G. mellonella* Output**				
TTTT	G4h 1-1	55408	44974	10434
GAAG	G4h 1-2	7360	5887	1473
ACCA	G4h 1-3	58672	47612	11060
CTTA	G4h 2-1	1442	1189	253
ACGT	G4h 2-2	8225	6703	1522
CCTT	G4h 2-3	49255	39922	9333
AGGA	G4h 3-1	52377	42437	9940
AACC	G4h 3-2	56691	45964	10727
AGTC	G4h 3-3	52605	42637	9968

a: The gray background samples reads < 50% of the total insertion positions.

Supplementary Table 3. Virulence factors in *G. mellonella* model found by Tn-seq.[a]

Gene ID	Gene	Product	Notes on function and known links to virulence	log(G4h/T0) AVG
**Energy production and conversion**				
K43816_GM000319	*glpD*	Glycerol-3-phosphate dehydrogenase	Low-capsule, Kpn (Dorman et al., 2018), *Listeria monocytogenes* (Crespo Tapia et al., 2018) (Koomen et al., 2018)	-1.932
K43816_GM000767	*ubiI*	2-polyprenyl-6-methoxyphenol hydroxylase and related FAD-dependent oxidoreductases	UPEC (Floyd et al., 2016)	-1.752
K43816_GM003140	*ndh*	NADH dehydrogenase, FAD-containing subunit	(Lencina et al., 2018;Vilcheze et al., 2018)	-2.165
K43816_GM004572	*ppa*	Inorganic pyrophosphatase	(Wu et al., 2010;Galizzi et al., 2013)	-3.549
K43816_GM005032	*atpC*	FoF1-type ATP synthase, epsilon subunit	*Streptococcus pneumoniae* (Cortes et al., 2008)	-1.972
**Cell cycle control, cell division, chromosome partitioning**				
K43816_GM000281	*ftsX*	Cell division protein	*Fusobacterium nucleatum* (Wu et al., 2018)	-3.142
K43816_GM003151	*yceG*	Cell division protein, involved in septum cleavage	*Bacillus anthracis* (Franks et al., 2014)	-2.415
K43816_GM004949	*ftsN*	Cell division protein	(Aurass et al., 2016)	-1.950
**Amino acid transport and metabolism**				
K43816_GM000351	*aroB*	3-dehydroquinate synthetase	Vaccine candidate (Stritzker et al., 2004;Cuccui et al., 2007;Robinson et al., 2015)	-2.562
K43816_GM001022	*cysH*	3'-phosphoadenosine 5'-phosphosulfate sulfotransferase (PAPS reductase)/FAD synthetase or related enzyme	*Vibrio fischeri* (Singh et al., 2015)	-3.014
K43816_GM001410	*aroC*	Chorismate synthase	Vaccine candidate (Xiong et al., 2015;Liu et al., 2018)	-2.824
K43816_GM002443	*fliY*	ABC-type amino acid transport/signal transduction system, periplasmic component/domain	Flagella (Schuhmacher et al., 2015)	-1.902
K43816_GM003319	*aroA*	5-enolpyruvylshikimate-3-phosphate synthase	(Karki and Ham, 2014)	-2.504
K43816_GM003532	*aroG*	3-deoxy-D-arabino-heptulosonate 7-phosphate (DAHP) synthase	(Aruni et al., 2013)	-2.396
K43816_GM004876	*dapF*	Diaminopimelate epimerase	peptidoglycan synthesis (Liechti et al., 2018)	-2.665
**Nucleotide transport and metabolism (purine and pyrimidine metabolism)**				
K43816_GM000125	*pyrE*	Orotate phosphoribosyltransferase	(Yuan et al., 2013)	-2.294
K43816_GM001322	*purC*	Phosphoribosylaminoimidazole-succinocarboxamide synthase	(Yuan et al., 2013)	-3.787
K43816_GM001487	*nrdB*	Ribonucleotide reductase beta subunit, ferritin-like domain	(Yuan et al., 2013)	-1.790
K43816_GM002960	*pyrF*	Orotidine-5'-phosphate decarboxylase	(Yuan et al., 2013)	-2.945
K43816_GM003172	*pyrC*	Dihydroorotase	(Yuan et al., 2013)	-3.303
K43816_GM003285	*pyrD*	Dihydroorotate dehydrogenase	(Schwager et al., 2013)	-3.613
K43816_GM003815	*purE*	Phosphoribosylcarboxyaminoimidazole (NCAIR) mutase	(Yuan et al., 2013)	-3.819
K43816_GM004266	*carA*	Carbamoylphosphate synthase small subunit	(Yuan et al., 2013)	-2.085
K43816_GM004879	*cyaA*	Adenylate cyclase	(Lin et al., 2013)	-2.534

Continued from Supplementary Table 3

**Carbohydrate transport and metabolism**				
K43816_GM000751	*tkt*	Transketolase	(Kovarova et al., 2018)	-2.457
K43816_GM000887	*araE*	Predicted arabinose efflux permease		-2.216
K43816_GM001632	*mshA*	Group 1 glycosyl transferase	Component of capsule biosynthesis operon	-2.965
K43816_GM001633	*orf8*	Glycogen synthase	Component of capsule biosynthesis operon (Shu et al., 2009)	-1.796
K43816_GM001642	*manB*	Phosphomannomutase	Component of capsule biosynthesis operon (Shu et al., 2009)	-1.833
K43816_GM001876	*zwf*	Glucose-6-phosphate 1-dehydrogenase	(Guo et al., 2015)	-2.432
**Coenzyme transport and metabolism (Vitamin B6 metabolism)**				
K43816_GM001228	*pdxJ*	Pyridoxine 5'-phosphate synthase	(Grubman et al., 2010)	-2.851
K43816_GM001420	*pdxB*	Phosphoglycerate dehydrogenase or related dehydrogenase	(Grubman et al., 2010)	-2.622
K43816_GM002289	*pdxH*	Pyridoxine/pyridoxamine 5'-phosphate oxidase	(Grubman et al., 2010)	-3.070
K43816_GM003320	*SerC (pdxC)*	Phosphoserine aminotransferase	(Descamps et al., 2018)	-3.354
K43816_GM004257	*pdxA*	4-hydroxy-L-threonine phosphate dehydrogenase	(Grubman et al., 2010)	-2.601
**Lipid transport and metabolism**				
K43816_GM003153	*fabF*	3-oxoacyl-(acyl-carrier-protein) synthase	(Li et al., 2023)	-2.189
**Translation, ribosomal structure and biogenesis**				
K43816_GM000425	*def*	Peptide deformylase	Essential gene	-2.631
K43816_GM000532	*rbfA*	Ribosome-binding factor A		-2.351
K43816_GM000536	*pnp*	Polyribonucleotide nucleotidyltransferase (polynucleotide phosphorylase)	(Rosenzweig and Schesser, 2007)	-1.868
K43816_GM001202	*rluD* (*vagC*)	Pseudouridylate synthase, 23S rRNA- or tRNA-specific	*Y. pestis* (Garbom et al., 2004)	-2.604
K43816_GM004218	*mraW*	16S rRNA C1402 N4-methylase RsmH	*E. coli* (Xu et al., 2019)	-2.086
**Transcription**				
K43816_GM000977	*gcvA*	DNA-binding transcriptional regulator		-3.004
K43816_GM001936	*fadR* (*YijC*)	DNA-binding transcriptional regulator, FadR family	EHEC, *V. cholerae* (Binepal et al., 2016;Pifer et al., 2018)	-1.859
K43816_GM002106	*mexL*	DNA-binding transcriptional regulator, AcrR family	*P. aeruginosa* (Yu et al., 2025)	-1.988
K43816_GM002537	*gntR_1*	DNA-binding transcriptional regulator, LacI/PurR family	(Haine et al., 2005;Li et al., 2017)	-2.391
K43816_GM002807	*dmlR*	DNA-binding transcriptional regulator, LysR family		-1.864
K43816_GM002967	*cysB*	DNA-binding transcriptional regulator, LysR family	(Song et al., 2019)	-4.209
K43816_GM003718	*yjiR*	DNA-binding transcriptional regulator, MocR family, contains an aminotransferase domain		-1.790
K43816_GM004010	*ecpR*	DNA-binding transcriptional regulator, CsgD family		-1.914
K43816_GM004307	*arcA*	DNA-binding response regulator, OmpR family, contains REC and winged-helix (wHTH) domain	(Merrell, 2007;Buettner et al., 2008)	-1.945
K43816_GM004609	*ulaR*	DNA-binding transcriptional regulator of sugar metabolism, DeoR/GlpR family		-2.414

Continued from Supplementary Table 3				
K43816_GM004765	*lexA*	SOS-response transcriptional repressor LexA (RecA-mediated autopeptidase)	(Walter et al., 2015)	-2.409
K43816_GM004925	*fabR*	DNA-binding transcriptional regulator, AcrR family	(Hermans et al., 2016)	-2.950
K43816_GM004945	*metJ*	Transcriptional regulator of met regulon	(Bourgeois et al., 2018)	-3.822
**Replication, recombination and repair**				
K43816_GM000921	*recC*	Exonuclease V gamma subunit	(Tenor et al., 2004)	-2.738
K43816_GM001216	*srmB*	Superfamily II DNA and RNA helicase		-1.864
K43816_GM004490	-	Integrase		-1.761
K43816_GM004491	*wzyE*	Integrase	LPS bioynthesis (Shivatare et al., 2022)	-1.744
K43816_GM004872	*uvrD*	Superfamily I DNA or RNA helicase		-1.764
K43816_GM004874	*xerC*	Site-specific recombinase	(Atwood et al., 2016)	-2.711
K43816_GM004888	*wzy*	ECA polymerase	(Nath and Morona, 2015;Nath et al., 2015)	-2.991
K43816_GM005000	*polA*	DNA polymerase I - 3'-5' exonuclease and polymerase domains		-3.307
**Cell wall/membrane/envelope biogenesis**				
K43816_GM000140	*WaaQ (rfaQ)*	ADP-heptose, LPS heptosyltransferase	LPS bioynthesis	-2.446
K43816_GM000141	*wabN*	Peptidoglycan / xylan / chitin deacetylase	LPS bioynthesis	-3.613
K43816_GM000498	*lptB*	ABC-type lipopolysaccharide export system, ATPase component	(Sperandeo et al., 2007;Martorana et al., 2016)	-1.817
K43816_GM000500	*lptC*	Lipopolysaccharide export system protein	(Sestito et al., 2014)	-4.130
K43816_GM000652	*tolC*	Outer membrane protein	(Gil et al., 2006)	-2.656
K43816_GM001276	*BamB (yfgL)*	Outer membrane protein assembly factor BamB, contains PQQ-like beta-propeller repeat	(Fardini et al., 2007;Hsieh et al., 2016)	-3.308
K43816_GM001626	*wzi*	Outer membrane protein Wzi involved in capsule attachment	Kpn(Shu et al., 2009)	-2.515
K43816_GM001627	*wza*	Periplasmic protein involved in polysaccharide export, contains SLBB domain of the beta-grasp fold	Kpn(Shu et al., 2009)	-4.189
K43816_GM002042	*galU*	UTP-glucose-1-phosphate uridylyltransferase	(Chang et al., 1996)	-3.079
K43816_GM002961	*lapB (YciM)*	Lipopolysaccharide biosynthesis regulator YciM, contains six TPR domains and a predicted metal-binding C-terminal domain	wzy	-2.039
K43816_GM003617	*dacC*	D-alanyl-D-alanine carboxypeptidase	(Zarantonelli et al., 2013)	-3.223
K43816_GM004449	*epsI*	Exopolysaccharide biosynthesis protein EpsI, predicted pyruvyl transferase	(Yanez et al., 2008)	-1.806
K43816_GM004891	*wecE*	dTDP-4-amino-4,6-dideoxygalactose transaminase	enterobacterial common antigen (ECA) biosynthesis	-2.013
K43816_GM004893	*rfbA*	dTDP-glucose pyrophosphorylase,	(Guo et al., 2012), O-antigen export system permease protein	-2.034
K43816_GM004894	*rfbB*	dTDP-D-glucose 4,6-dehydratase	the synthesis of O-specific LPS	-3.868
K43816_GM004898	*wecA*	UDP-N-acetylmuramyl pentapeptide phosphotransferase/UDP-N-acetylglucosamine-1-phosphate transferase	capsule biosynthesis/regulation	-1.906
**Post translational modification, protein turnover, chaperones**				
K43816_GM000480	*sspA*	Stringent starvation protein A, Glutathione S-transferase	Francisella. EHEC. Etc. (Cuthbert et al., 2017)	-2.456

Continued from Supplementary Table 3

K43816_GM000784	*ygfY (SdhE)*	Succinate dehydrogenase flavin-adding protein, antitoxin component of the CptAB toxin-antitoxin module	(Masuda et al., 2012;McNeil et al., 2014)	-1.925
K43816_GM001177	*smpB*	SsrA tmRNA-binding protein	High-capsule	-1.853
K43816_GM001899	*prc*	C-terminal processing protease CtpA/Prc, contains a PDZ domain	(Seo and Darwin, 2013)	-1.881
K43816_GM001933	*dsbB*	Disulfide bond formation protein DsbB	Burkholderia pseudomallei(McMahon et al., 2018)	-4.408
K43816_GM002695	*yedK*	Putative SOS response-associated peptidase YedK		-2.312
K43816_GM003895	*lon*	ATP-dependent Lon protease, bacterial type	(Takaya et al., 2003;Van Melderen and Aertsen, 2009;Lee et al., 2018)	-1.761
K43816_GM004620	*hflC*	Regulator of protease activity HflC, stomatin/prohibitin superfamily		-2.010
K43816_GM004621	*hflK*	Regulator of protease activity HflK, stomatin/prohibitin superfamily		-1.964
K43816_GM004950	*hslV*	ATP-dependent protease HslVU (ClpYQ), peptidase subunit	(Dong et al., 2017)	-1.878
K43816_GM005003	*dsbA*	Protein-disulfide isomerase	(Denoncin et al., 2010;Vilches et al., 2012)	-4.097
**Inorganic ion transport and metabolism**				
K43816_GM000422	*trkA*	Trk K+ transport system, NAD-binding component	(Alkhuder et al., 2010;Binepal et al., 2016)	-2.291
K43816_GM001869	*znuB*	ABC-type Mn2+/Zn2+ transport system, permease component	(Wang et al., 2014)	-2.031
K43816_GM002570		Alkylhydroperoxidase family enzyme, contains CxxC motif		-1.773
K43816_GM003676	*fepB*	ABC-type Fe2+-enterobactin transport system, periplasmic component	(Palacios et al., 2017)	-2.823
K43816_GM004584	*cysQ*	3'-Phosphoadenosine 5'-phosphosulfate (PAPS) 3'-phosphatase	(Laasik et al., 2005;Gebhardt et al., 2015)	-2.028
K43816_GM004840	*trkG*	Trk-type K+ transport system, membrane component	(Alkhuder et al., 2010;Binepal et al., 2016)	-2.514
**General function prediction only**				
K43816_GM001285	*guaB*	NAD(P)H-dependent flavin oxidoreductase YrpB, nitropropane dioxygenase family	(Santiago et al., 2015;Kofoed et al., 2016)	-4.314
K43816_GM001315	*bepA (yfgC)*	Putative Zn-dependent protease, contains TPR repeats	(Daimon et al., 2017)	-2.938
K43816_GM002549	*ahlK*	Glyoxylase or a related metal-dependent hydrolase, beta-lactamase superfamily II		-1.845
K43816_GM003841	*ybaB*	Conserved DNA-binding protein YbaB		-1.801
K43816_GM000454	*yhdP (wzy)*	outer membrane permeability factor YhdP	LPS bioynthesis	-2.370
K43816_GM001872	*mepM*	Cell envelope opacity-associated protein A	UPEC(Huang et al., 2024)	-1.953
K43816_GM002502	*YhfF (prkB)*	putative phosphoribulokinase		-2.024
K43816_GM003311	*ycaR*	Uncharacterized conserved protein YbaR, Trm112 family，UPF0434 family protein YcaR		-2.680
K43816_GM003902	*yajG*	Uncharacterized lipoprotein YajG		-2.684
K43816_GM004877	*yifL*	Predicted small periplasmic lipoprotein YifL		-5.143
K43816_GM003596	*phoH*	Phosphate starvation-inducible protein PhoH, predicted ATPase	*S. Typhimurium*(Valdespino-Díaz et al., 2022)	-2.635
**Intracellular trafficking, secretion, and vesicular transport**				
K43816_GM002634	*hcp*	Type VI protein secretion system component Hcp (secreted cytotoxin)	(Kim et al., 2017;Ma et al., 2017)	-1.855

Continued from Supplementary Table 3				
K43816_GM003939	*secD*	Preprotein translocase subunit SecD	(Matsuyama et al., 1993)	-3.724
K43816_GM004849	*tatC*	Sec-independent protein secretion pathway component TatC	(Ding and Christie, 2003;De Buck et al., 2005)	-1.920
K43816_GM004850	*tatB*	Sec-independent protein translocase protein	(Ding and Christie, 2003;De Buck et al., 2005)	-2.662
K43816_GM004851	*tatA*	Sec-independent protein translocase protein	(Ding and Christie, 2003)	-3.178
K43816_GM003850	*acrA*	Multidrug efflux pump subunit (membrane-fusion protein)	(Blair et al., 2009)	-2.631
K43816_GM003851	*acrB*	Multidrug efflux pump subunit	(Blair et al., 2009)	-2.376
**Absent from COG database**				
K43816_GM001122		hypothetical protein		-1.965
K43816_GM001195		hypothetical protein		-2.382
K43816_GM001288		hypothetical protein		-2.561
K43816_GM001521		hypothetical protein		-1.822
K43816_GM001638		hypothetical protein		-2.792
K43816_GM001720		hypothetical protein		-1.926
K43816_GM001807		hypothetical protein		-2.212
K43816_GM001831		hypothetical protein		-2.386
K43816_GM001838		hypothetical protein		-2.514
K43816_GM002429		hypothetical protein		-1.919
K43816_GM002451		hypothetical protein		-2.746
K43816_GM002835		hypothetical protein		-1.732
K43816_GM003174		hypothetical protein		-1.892
K43816_GM003179		hypothetical protein		-2.439
K43816_GM003678		hypothetical protein		-1.794
K43816_GM003900		hypothetical protein		-1.924
K43816_GM004337		hypothetical protein		-2.153
K43816_GM004464		hypothetical protein		-3.030
K43816_GM004474		hypothetical protein		-1.891
K43816_GM004494		hypothetical protein		-1.801

a: The gray blackground genes are vital genes that are exclusively present in the vivo group of the *G. mellonella*, but not in the LB group *in vitro*.

Supplementary References

Alkhuder, K., Meibom, K.L., Dubail, I., Dupuis, M., and Charbit, A. (2010). Identification of *trkH*, encoding a potassium uptake protein required for *Francisella tularensis* systemic dissemination in mice. *PLoS One* 5**,** e8966.

Aruni, A.W., Robles, A., and Fletcher, H.M. (2013). VimA mediates multiple functions that control virulence in *Porphyromonas gingivalis*. *Mol Oral Microbiol* 28**,** 167-180.

Atwood, D.N., Beenken, K.E., Loughran, A.J., Meeker, D.G., Lantz, T.L., Graham, J.W., Spencer, H.J., and Smeltzer, M.S. (2016). XerC Contributes to Diverse Forms of *Staphylococcus aureus* Infection via agr-Dependent and *agr*-Independent Pathways. *Infect Immun* 84**,** 1214-1225.

Aurass, P., Gerlach, T., Becher, D., Voigt, B., Karste, S., Bernhardt, J., Riedel, K., Hecker, M., and Flieger, A. (2016). Life Stage-specific Proteomes of *Legionella pneumophila* Reveal a Highly Differential Abundance of Virulence-associated Dot/Icm effectors. *Mol Cell Proteomics* 15**,** 177-200.

Binepal, G., Gill, K., Crowley, P., Cordova, M., Brady, L.J., Senadheera, D.B., and Cvitkovitch, D.G. (2016). Trk2 Potassium Transport System in *Streptococcus* mutans and Its Role in Potassium Homeostasis, Biofilm Formation, and Stress Tolerance. *J Bacteriol* 198**,** 1087-1100.

Blair, J.M., La Ragione, R.M., Woodward, M.J., and Piddock, L.J. (2009). Periplasmic adaptor protein AcrA has a distinct role in the antibiotic resistance and virulence of *Salmonella enterica* serovar Typhimurium. *J Antimicrob Chemother* 64**,** 965-972.

Bourgeois, J.S., Zhou, D., Thurston, T.L.M., Gilchrist, J.J., and Ko, D.C. (2018). Methylthioadenosine Suppresses *Salmonella* Virulence. *Infect Immun* 86, e00429-18.

Buettner, F.F., Maas, A., and Gerlach, G.F. (2008). An *Actinobacillus pleuropneumoniae arcA* deletion mutant is attenuated and deficient in biofilm formation. *Vet Microbiol* 127**,** 106-115.

Chang, H.Y., Lee, J.H., Deng, W.L., Fu, T.F., and Peng, H.L. (1996). Virulence and outer membrane properties of a *galU* mutant of *Klebsiella pneumoniae* CG43. *Microb Pathog* 20**,** 255-261.

Cortes, P.R., Orio, A.G., Regueira, M., Piñas, G.E., and Echenique, J. (2008). Characterization of *in vitro*-generated and clinical optochin-resistant strains of *Streptococcus pneumoniae* isolated from Argentina. *J Clin Microbiol* 46**,** 1930-1934.

Crespo Tapia, N., Den Besten, H.M.W., and Abee, T. (2018). Glycerol metabolism induces *Listeria monocytogenes* biofilm formation at the air-liquid interface. *Int J Food Microbiol* 273**,** 20-27.

Cuccui, J., Easton, A., Chu, K.K., Bancroft, G.J., Oyston, P.C., Titball, R.W., and Wren, B.W. (2007). Development of signature-tagged mutagenesis in *Burkholderia pseudomallei* to identify genes important in survival and pathogenesis. *Infect Immun* 75**,** 1186-1195.

Cuthbert, B.J., Ross, W., Rohlfing, A.E., Dove, S.L., Gourse, R.L., Brennan, R.G., and Schumacher, M.A. (2017). Dissection of the molecular circuitry controlling virulence in *Francisella tularensis*. *Genes Dev* 31**,** 1549-1560.

Daimon, Y., Iwama-Masui, C., Tanaka, Y., Shiota, T., Suzuki, T., Miyazaki, R., Sakurada, H., and Lithgow, T. (2017). The TPR domain of BepA is required for productive interaction with substrate proteins and the beta-barrel assembly machinery complex. 106**,** 760-776.

De Buck, E., Maes, L., Meyen, E., Van Mellaert, L., Geukens, N., Anne, J., and Lammertyn, E. (2005). *Legionella pneumophila* Philadelphia-1 *tatB* and *tatC* affect intracellular replication and biofilm formation. *Biochem Biophys Res Commun* 331**,** 1413-1420.

Denoncin, K., Vertommen, D., Paek, E., and Collet, J.F. (2010). The protein-disulfide isomerase DsbC cooperates with SurA and DsbA in the assembly of the essential beta-barrel protein LptD. *J Biol Chem* 285**,** 29425-29433.

Descamps, T., De Smet, L., De Vos, P., and De Graaf, D.C. (2018). Unbiased random mutagenesis contributes to a better understanding of the virulent behaviour of *Paenibacillus* larvae. *J Appl Microbiol* 124**,** 28-41.

Ding, Z., and Christie, P.J. (2003). *Agrobacterium tumefaciens* twin-arginine-dependent translocation is important for virulence, flagellation, and chemotaxis but not type IV secretion. *J Bacteriol* 185**,** 760-771.

Dong, S.L., Hu, W.L., Ge, Y.M., Ojcius, D.M., Lin, X., and Yan, J. (2017). A leptospiral AAA+ chaperone-Ntn peptidase complex, HslUV, contributes to the intracellular survival of *Leptospira interrogans* in hosts and the transmission of leptospirosis. *Emerg Microbes Infect* 6**,** e105.

Dorman, M.J., Feltwell, T., Goulding, D.A., Parkhill, J., and Short, F.L. (2018). The Capsule Regulatory Network of *Klebsiella pneumoniae* Defined by density-TraDISort. *MBio* 9(6):e01863-18.

Fardini, Y., Chettab, K., Grepinet, O., Rochereau, S., Trotereau, J., Harvey, P., Amy, M., Bottreau, E., Bumstead, N., Barrow, P.A., and Virlogeux-Payant, I. (2007). The YfgL lipoprotein is essential for type III secretion system expression and virulence of *Salmonella enterica Serovar Enteritidis*. *Infect Immun* 75**,** 358-370.

Floyd, K.A., Mitchell, C.A., Eberly, A.R., Colling, S.J., Zhang, E.W., Depas, W., Chapman, M.R., Conover, M., Rogers, B.R., Hultgren, S.J., and Hadjifrangiskou, M. (2016). The UbiI (VisC) Aerobic Ubiquinone Synthase Is Required for Expression of Type 1 Pili, Biofilm Formation, and Pathogenesis in Uropathogenic *Escherichia coli*. *J Bacteriol* 198**,** 2662-2672.

Franks, S.E., Ebrahimi, C., Hollands, A., Okumura, C.Y., Aroian, R.V., Nizet, V., and Mcgillivray, S.M. (2014). Novel role for the *yceGH* tellurite resistance genes in the pathogenesis of *Bacillus anthracis*. *Infect Immun* 82**,** 1132-1140.

Galizzi, M., Bustamante, J.M., Fang, J., Miranda, K., Soares Medeiros, L.C., Tarleton, R.L., and Docampo, R. (2013). Evidence for the role of vacuolar soluble pyrophosphatase and inorganic polyphosphate in *Trypanosoma cruzi* persistence. *Mol Microbiol* 90**,** 699-715.

Garbom, S., Forsberg, A., Wolf-Watz, H., and Kihlberg, B.M. (2004). Identification of novel virulence-associated genes via genome analysis of hypothetical genes. *Infect Immun* 72**,** 1333-1340.

Gebhardt, M.J., Gallagher, L.A., Jacobson, R.K., Usacheva, E.A., Peterson, L.R., Zurawski, D.V., and Shuman, H.A. (2015). Joint Transcriptional Control of Virulence and Resistance to Antibiotic and Environmental Stress in *Acinetobacter baumannii*. *MBio* 6**,** e01660-01615.

Gil, H., Platz, G.J., Forestal, C.A., Monfett, M., Bakshi, C.S., Sellati, T.J., Furie, M.B., Benach, J.L., and Thanassi, D.G. (2006). Deletion of TolC orthologs in *Francisella tularensis* identifies roles in multidrug resistance and virulence. *Proc Natl Acad Sci U S A* 103**,** 12897-12902.

Grubman, A., Phillips, A., Thibonnier, M., Kaparakis-Liaskos, M., Johnson, C., Thiberge, J.M., Radcliff, F.J., Ecobichon, C., Labigne, A., De Reuse, H., Mendz, G.L., and Ferrero, R.L. (2010). Vitamin B6 is required for full motility and virulence in *Helicobacter pylori*. *MBio* 1(3):e00112-10.

Guo, W., Cui, Y.P., Li, Y.R., Che, Y.Z., Yuan, L., Zou, L.F., Zou, H.S., and Chen, G.Y. (2012). Identification of seven *Xanthomonas oryzae* pv. *oryzicola* genes potentially involved in pathogenesis in rice. *Microbiology* 158**,** 505-518.

Guo, W., Zou, L.F., Cai, L.L., and Chen, G.Y. (2015). Glucose-6-phosphate dehydrogenase is required for extracellular polysaccharide production, cell motility and the full virulence of *Xanthomonas oryzae* pv. *oryzicola*. *Microb Pathog* 78**,** 87-94.

Haine, V., Sinon, A., Van Steen, F., Rousseau, S., Dozot, M., Lestrate, P., Lambert, C., Letesson, J.J., and De Bolle, X. (2005). Systematic targeted mutagenesis of *Brucella melitensis* 16M reveals a major role for GntR regulators in the control of virulence. *Infect Immun* 73**,** 5578-5586.

Hermans, K., Roberfroid, S., Thijs, I.M., Kint, G., De Coster, D., Marchal, K., Vanderleyden, J., De Keersmaecker, S.C., and Steenackers, H.P. (2016). FabR regulates *Salmonella* biofilm formation via its direct target FabB. *BMC Genomics* 17**,** 253.

Hsieh, P.F., Hsu, C.R., Chen, C.T., Lin, T.L., and Wang, J.T. (2016). The *Klebsiella pneumoniae* YfgL (BamB) lipoprotein contributes to outer membrane protein biogenesis, type-1 fimbriae expression, anti-phagocytosis, and in vivo virulence. *Virulence* 7**,** 587-601.

Huang, W.C., Dwija, I., Hashimoto, M., Wu, J.J., Wang, M.C., Kao, C.Y., Lin, W.H., Wang, S., and Teng, C.H. (2024). Peptidoglycan endopeptidase MepM of uropathogenic *Escherichia coli* contributes to competitive fitness during urinary tract infections. *BMC Microbiol* 24**,** 190.

Karki, H.S., and Ham, J.H. (2014). The roles of the shikimate pathway genes, *aroA* and *aroB*, in virulence, growth and UV tolerance of *Burkholderia glumae* strain 411gr-6. *Mol Plant Pathol* 15**,** 940-947.

Kim, J., Lee, J.Y., Lee, H., Choi, J.Y., Kim, D.H., Wi, Y.M., Peck, K.R., and Ko, K.S. (2017). Microbiological features and clinical impact of the type VI secretion system (T6SS) in *Acinetobacter baumannii* isolates causing bacteremia. *Virulence* 8**,** 1378-1389.

Kofoed, E.M., Yan, D., Katakam, A.K., Reichelt, M., Lin, B., Kim, J., Park, S., Date, S.V., Monk, I.R., Xu, M., Austin, C.D., Maurer, T., and Tan, M.W. (2016). De Novo Guanine Biosynthesis but Not the Riboswitch-Regulated Purine Salvage Pathway Is Required for *Staphylococcus aureus* Infection In Vivo. *J Bacteriol* 198**,** 2001-2015.

Koomen, J., Den Besten, H.M.W., Metselaar, K.I., Tempelaars, M.H., Wijnands, L.M., Zwietering, M.H., and Abee, T. (2018). Gene profiling-based phenotyping for identification of cellular parameters that contribute to fitness, stress-tolerance and virulence of *Listeria monocytogenes* variants. *Int J Food Microbiol* 283**,** 14-21.

Kovarova, J., Pountain, A.W., Wildridge, D., Weidt, S., Bringaud, F., Burchmore, R.J.S., Achcar, F., and Barrett, M.P. (2018). Deletion of transketolase triggers a stringent metabolic response in promastigotes and loss of virulence in amastigotes of *Leishmania mexicana*. *PLoS Pathog* 14**,** e1006953.

Laasik, E., Ojarand, M., Pajunen, M., Savilahti, H., and Mae, A. (2005). Novel mutants of *Erwinia carotovora subsp. carotovora* defective in the production of plant cell wall degrading

enzymes generated by Mu transpososome-mediated insertion mutagenesis. *FEMS Microbiol Lett* 243**,** 93-99.

Lee, J.H., Ancona, V., and Zhao, Y. (2018). Lon protease modulates virulence traits in *Erwinia amylovora* by direct monitoring of major regulators and indirectly through the Rcs and Gac-Csr regulatory systems. *Mol Plant Pathol* 19**,** 827-840.

Lencina, A.M., Franza, T., Sullivan, M.J., Ulett, G.C., Ipe, D.S., Gaudu, P., Gennis, R.B., and Schurig-Briccio, L.A. (2018). Type 2 NADH Dehydrogenase Is the Only Point of Entry for Electrons into the *Streptococcus agalactiae* Respiratory Chain and Is a Potential Drug Target. *MBio* 9(4):e01034-18.

Li, J., Yan, B., He, B., Li, L., Zhou, X., Wu, N., Wang, Q., Guo, X., Zhu, T., and Qin, J. (2023). Development of phage resistance in multidrug-resistant K*lebsiella pneumoniae* is associated with reduced virulence: a case report of a personalised phage therapy. *Clin Microbiol Infect* 29**,** 1601.e1601-1601.e1607.

Li, Z.Q., Zhang, J.L., Xi, L., Yang, G.L., Wang, S.L., Zhang, X.G., Zhang, J.B., and Zhang, H. (2017). Deletion of the transcriptional regulator GntR down regulated the expression of Genes Related to Virulence and Conferred Protection against Wild-Type *Brucella* Challenge in BALB/c Mice. *Mol Immunol* 92**,** 99-105.

Liechti, G., Singh, R., Rossi, P.L., Gray, M.D., Adams, N.E., and Maurelli, A.T. (2018). *Chlamydia trachomatis dapF* Encodes a Bifunctional Enzyme Capable of Both d-Glutamate Racemase and Diaminopimelate Epimerase Activities. *mBio* 9(2):e00204-18.

Lin, C.T., Chen, Y.C., Jinn, T.R., Wu, C.C., Hong, Y.M., and Wu, W.H. (2013). Role of the cAMP-dependent carbon catabolite repression in capsular polysaccharide biosynthesis in *Klebsiella pneumoniae*. *PLoS One* 8**,** e54430.

Liu, X., Jiao, C., Ma, Y., Wang, Q., and Zhang, Y. (2018). A live attenuated *Vibrio anguillarum* vaccine induces efficient immunoprotection in Tiger puffer (Takifugu rubripes). *Vaccine* 36**,** 1460-1466.

Ma, J., Pan, Z., Huang, J., Sun, M., Lu, C., and Yao, H. (2017). The Hcp proteins fused with diverse extended-toxin domains represent a novel pattern of antibacterial effectors in type VI secretion systems. *Virulence* 8**,** 1189-1202.

Martorana, A.M., Benedet, M., Maccagni, E.A., Sperandeo, P., Villa, R., Deho, G., and Polissi, A. (2016). Functional Interaction between the Cytoplasmic ABC Protein LptB and the Inner Membrane LptC Protein, Components of the Lipopolysaccharide Transport Machinery in *Escherichia coli*. *J Bacteriol* 198**,** 2192-2203.

Masuda, H., Tan, Q., Awano, N., Yamaguchi, Y., and Inouye, M. (2012). A novel membrane-bound toxin for cell division, CptA (YgfX), inhibits polymerization of cytoskeleton proteins, FtsZ and MreB, in *Escherichia coli*. *FEMS Microbiol Lett* 328**,** 174-181.

Matsuyama, S., Fujita, Y., and Mizushima, S. (1993). SecD is involved in the release of translocated secretory proteins from the cytoplasmic membrane of *Escherichia coli*. *Embo j* 12**,** 265-270.

Mcmahon, R.M., Ireland, P.M., Sarovich, D.S., Petit, G., Jenkins, C.H., Sarkar-Tyson, M., Currie, B.J., and Martin, J.L. (2018). Virulence of the Melioidosis Pathogen *Burkholderia pseudomallei* Requires the Oxidoreductase Membrane Protein DsbB. *Infect Immun* 86, e00938-17.

Mcneil, M.B., Hampton, H.G., Hards, K.J., Watson, B.N., Cook, G.M., and Fineran, P.C. (2014). The succinate dehydrogenase assembly factor, SdhE, is required for the flavinylation and activation of fumarate reductase in bacteria. *FEBS Lett* 588**,** 414-421.

Merrell, D.S. (2007). Environmental stress regulates *Shigella* virulence: interplay between anerobiosis and iron acquisition. *Future Microbiol* 2**,** 601-604.

Nath, P., and Morona, R. (2015). Mutational analysis of the major periplasmic loops of *Shigella flexneri* Wzy: identification of the residues affecting O antigen modal chain length control, and Wzz-dependent polymerization activity. *Microbiology* 161**,** 774-785.

Nath, P., Tran, E.N., and Morona, R. (2015). Mutational analysis of the *Shigella flexneri* O-antigen polymerase Wzy: identification of Wzz-dependent Wzy mutants. *J Bacteriol* 197**,** 108-119.

Palacios, M., Broberg, C.A., Walker, K.A., and Miller, V.L. (2017). A Serendipitous Mutation Reveals the Severe Virulence Defect of a *Klebsiella pneumoniae fepB* Mutant. *mSphere* 2, e00341-17.

Pifer, R., Russell, R.M., Kumar, A., Curtis, M.M., and Sperandio, V. (2018). Redox, amino acid, and fatty acid metabolism intersect with bacterial virulence in the gut. *Proc Natl Acad Sci U S A* 115**,** E10712-e10719.

Robinson, C., Heather, Z., Slater, J., Potts, N., Steward, K.F., Maskell, D.J., Fontaine, M.C., Lee, J.J., Smith, K., and Waller, A.S. (2015). Vaccination with a live multi-gene deletion strain protects horses against virulent challenge with *Streptococcus equi*. *Vaccine* 33**,** 1160-1167.

Rosenzweig, J.A., and Schesser, K. (2007). Polynucleotide phosphorylase and the T3SS. *Adv Exp Med Biol* 603**,** 217-224.

Santiago, A.E., Mann, B.J., Qin, A., Cunningham, A.L., Cole, L.E., Grassel, C., Vogel, S.N., Levine, M.M., and Barry, E.M. (2015). Characterization of *Francisella tularensis* Schu S4 defined mutants as live-attenuated vaccine candidates. *Pathog Dis* 73**,** ftv036.

Schuhmacher, J.S., Thormann, K.M., and Bange, G. (2015). How bacteria maintain location and number of flagella? *FEMS Microbiol Rev* 39**,** 812-822.

Schwager, S., Agnoli, K., Köthe, M., Feldmann, F., Givskov, M., Carlier, A., and Eberl, L. (2013). Identification of *Burkholderia cenocepacia* strain H111 virulence factors using nonmammalian infection hosts. *Infect Immun* 81**,** 143-153.

Seo, J., and Darwin, A.J. (2013). The *Pseudomonas aeruginosa* periplasmic protease CtpA can affect systems that impact its ability to mount both acute and chronic infections. *Infect Immun* 81**,** 4561-4570.

Sestito, S.E., Sperandeo, P., Santambrogio, C., Ciaramelli, C., Calabrese, V., Rovati, G.E., Zambelloni, L., Grandori, R., Polissi, A., and Peri, F. (2014). Functional characterization of *E. coli* LptC: interaction with LPS and a synthetic ligand. *Chembiochem* 15**,** 734-742.

Shivatare, S.S., Shivatare, V.S., and Wong, C.H. (2022). Glycoconjugates: Synthesis, Functional Studies, and Therapeutic Developments. *Chem Rev* 122**,** 15603-15671.

Shu, H.Y., Fung, C.P., Liu, Y.M., Wu, K.M., Chen, Y.T., Li, L.H., Liu, T.T., Kirby, R., and Tsai, S.F. (2009). Genetic diversity of capsular polysaccharide biosynthesis in *Klebsiella pneumoniae* clinical isolates. *Microbiology* 155**,** 4170-4183.

Singh, P., Brooks, J.F., 2nd, Ray, V.A., Mandel, M.J., and Visick, K.L. (2015). CysK Plays a Role in Biofilm Formation and Colonization by *Vibrio fischeri*. 81**,** 5223-5234.

Song, Y., Yang, C., Chen, G., Zhang, Y., Seng, Z., Cai, Z., Zhang, C., Yang, L., Gan, J., and Liang, H. (2019). Molecular insights into the master regulator CysB-mediated bacterial virulence in *Pseudomonas aeruginosa*. *Mol Microbiol* 111**,** 1195-1210.

Sperandeo, P., Cescutti, R., Villa, R., Di Benedetto, C., Candia, D., Deho, G., and Polissi, A. (2007). Characterization of *lptA* and *lptB*, two essential genes implicated in lipopolysaccharide transport to the outer membrane of *Escherichia coli*. *J Bacteriol* 189**,** 244-253.

Stritzker, J., Janda, J., Schoen, C., Taupp, M., Pilgrim, S., Gentschev, I., Schreier, P., Geginat, G., and Goebel, W. (2004). Growth, virulence, and immunogenicity of *Listeria monocytogenes* aro mutants. *Infect Immun* 72**,** 5622-5629.

Takaya, A., Suzuki, M., Matsui, H., Tomoyasu, T., Sashinami, H., Nakane, A., and Yamamoto, T. (2003). Lon, a stress-induced ATP-dependent protease, is critically important for systemic *Salmonella enterica* serovar typhimurium infection of mice. *Infect Immun* 71**,** 690-696.

Tenor, J.L., Mccormick, B.A., Ausubel, F.M., and Aballay, A. (2004). Caenorhabditis elegans-based screen identifies *Salmonella* virulence factors required for conserved host-pathogen interactions. *Curr Biol* 14**,** 1018-1024.

Valdespino-Díaz, M.A., Rosales-Reyes, R., De La Cruz, M.A., and Bustamante, V.H. (2022). Regulatory Evolution of the phoH Ancestral Gene in *Salmonella enterica* Serovar Typhimurium. *J Bacteriol* 204**,** e0058521.

Van Melderen, L., and Aertsen, A. (2009). Regulation and quality control by Lon-dependent proteolysis. *Res Microbiol* 160**,** 645-651.

Vilches, S., Jimenez, N., Merino, S., and Tomas, J.M. (2012). The *Aeromonas dsbA* mutation decreased their virulence by triggering type III secretion system but not flagella production. *Microb Pathog* 52**,** 130-139.

Vilcheze, C., Weinrick, B., Leung, L.W., and Jacobs, W.R., Jr. (2018). Plasticity of *Mycobacterium tuberculosis* NADH dehydrogenases and their role in virulence. *Proc Natl Acad Sci U S A* 115**,** 1599-1604.

Walter, B.M., Cartman, S.T., Minton, N.P., Butala, M., and Rupnik, M. (2015). The SOS Response Master Regulator LexA Is Associated with Sporulation, Motility and Biofilm Formation in *Clostridium difficile*. *PLoS One* 10**,** e0144763.

Wang, N., Ozer, E.A., Mandel, M.J., and Hauser, A.R. (2014). Genome-wide identification of *Acinetobacter baumannii* genes necessary for persistence in the lung. *MBio* 5**,** e01163-01114.

Wu, C., Al Mamun, A.a.M., Luong, T.T., Hu, B., Gu, J., Lee, J.H., D'amore, M., Das, A., and Ton-That, H. (2018). Forward Genetic Dissection of Biofilm Development by *Fusobacterium nucleatum*: Novel Functions of Cell Division Proteins FtsX and EnvC. *MBio* 9, e00360-18.

Wu, H.J., Seib, K.L., Srikhanta, Y.N., Edwards, J., Kidd, S.P., Maguire, T.L., Hamilton, A., Pan, K.T., Hsiao, H.H., Yao, C.W., Grimmond, S.M., Apicella, M.A., Mcewan, A.G., Wang, A.H., and Jennings, M.P. (2010). Manganese regulation of virulence factors and oxidative stress resistance in *Neisseria gonorrhoeae*. *J Proteomics* 73**,** 899-916.

Xiong, K., Chen, Z., Zhu, C., Li, J., Hu, X., Rao, X., and Cong, Y. (2015). Safety and immunogenicity of an attenuated *Salmonella enterica* serovar Paratyphi A vaccine candidate. *Int J Med Microbiol* 305**,** 563-571.

Xu, X., Zhang, H., Huang, Y., Zhang, Y., Wu, C., Gao, P., Teng, Z., Luo, X., Peng, X., Wang, X., Wang, D., Pu, J., Zhao, H., Lu, X., Lu, S., Ye, C., Dong, Y., Lan, R., and Xu, J. (2019). Beyond a Ribosomal RNA Methyltransferase, the Wider Role of MraW in DNA Methylation, Motility and Colonization in *Escherichia coli* O157:H7. *Front Microbiol* 10**,** 2520.

Yanez, M.E., Korotkov, K.V., Abendroth, J., and Hol, W.G. (2008). The crystal structure of a binary complex of two pseudopilins: EpsI and EpsJ from the type 2 secretion system of *Vibrio vulnificus*. *J Mol Biol* 375**,** 471-486.

Yu, Z., Wu, Z., Liu, D., Liu, H., Zhang, Y., Zheng, Y., Huang, Y., Liao, S., Wei, Y., Huang, W., Zhang, Z., Liu, X., Yu, H., Wang, D., Li, L., Long, F., and Ma, L.Z. (2025). Dual-function regulator MexL as a target to control phenazines production and pathogenesis of *Pseudomonas aeruginosa*. *Nat Commun* 16**,** 2000.

Yuan, Z., Wang, L., Sun, S., Wu, Y., and Qian, W. (2013). Genetic and proteomic analyses of a *Xanthomonas campestris* pv. *campestris purC* mutant deficient in purine biosynthesis and virulence. *J Genet Genomics* 40**,** 473-487.

Zarantonelli, M.L., Skoczynska, A., Antignac, A., El Ghachi, M., Deghmane, A.E., Szatanik, M., Mulet, C., Werts, C., Peduto, L., D'andon, M.F., Thouron, F., Nato, F., Lebourhis, L., Philpott, D.J., Girardin, S.E., Vives, F.L., Sansonetti, P., Eberl, G., Pedron, T., Taha, M.K., and Boneca, I.G. (2013). Penicillin resistance compromises Nod1-dependent proinflammatory activity and virulence fitness of *neisseria meningitidis*. *Cell Host Microbe* 13**,** 735-745.
